# Transcriptomic response to nitrogen availability reveals signatures of adaptive plasticity during tetraploid wheat domestication

**DOI:** 10.1093/plcell/koae202

**Published:** 2024-07-26

**Authors:** Alice Pieri, Romina Beleggia, Tania Gioia, Hao Tong, Valerio Di Vittori, Giulia Frascarelli, Elena Bitocchi, Laura Nanni, Elisa Bellucci, Fabio Fiorani, Nicola Pecchioni, Stefania Marzario, Concetta De Quattro, Antonina Rita Limongi, Pasquale De Vita, Marzia Rossato, Ulrich Schurr, Jacques L David, Zoran Nikoloski, Roberto Papa

**Affiliations:** Department of Agricultural, Food and Environmental Sciences, Marche Polytechnic University, via Brecce Bianche, Ancona 60131, Italy; Council for Agricultural Research and Economics (CREA), Research Centre for Cereal and Industrial Crops (CREA-CI), Foggia 71122, Italy; School of Agricultural, Forestry, Food and Environmental Sciences, University of Basilicata, Potenza 85100, Italy; Bioinformatics Department, Institute of Biochemistry and Biology, University of Potsdam, Potsdam 14476, Germany; Systems Biology and Mathematical Modeling Group, Max Planck Institute of Molecular Plant Physiology, Potsdam 14476, Germany; Department of Agricultural, Food and Environmental Sciences, Marche Polytechnic University, via Brecce Bianche, Ancona 60131, Italy; Department of Agricultural, Food and Environmental Sciences, Marche Polytechnic University, via Brecce Bianche, Ancona 60131, Italy; Department of Agricultural, Food and Environmental Sciences, Marche Polytechnic University, via Brecce Bianche, Ancona 60131, Italy; Department of Agricultural, Food and Environmental Sciences, Marche Polytechnic University, via Brecce Bianche, Ancona 60131, Italy; Department of Agricultural, Food and Environmental Sciences, Marche Polytechnic University, via Brecce Bianche, Ancona 60131, Italy; Institute of Biosciences and Geosciences (IBG-2): Plant Sciences, Forschungszentrum Julich GmbH, Julich 52428, Germany; Council for Agricultural Research and Economics (CREA), Research Centre for Cereal and Industrial Crops (CREA-CI), Foggia 71122, Italy; School of Agricultural, Forestry, Food and Environmental Sciences, University of Basilicata, Potenza 85100, Italy; Department of Biotechnology, University of Verona, Strada Le Grazie 15, Verona 37134, Italy; Department of Biotechnology, University of Verona, Strada Le Grazie 15, Verona 37134, Italy; Council for Agricultural Research and Economics (CREA), Research Centre for Cereal and Industrial Crops (CREA-CI), Foggia 71122, Italy; Department of Biotechnology, University of Verona, Strada Le Grazie 15, Verona 37134, Italy; Institute of Biosciences and Geosciences (IBG-2): Plant Sciences, Forschungszentrum Julich GmbH, Julich 52428, Germany; AGAP, Univ. Montpellier, CIRAD, INRAE, Institut Agro, Montpellier 34060, France; Bioinformatics Department, Institute of Biochemistry and Biology, University of Potsdam, Potsdam 14476, Germany; Systems Biology and Mathematical Modeling Group, Max Planck Institute of Molecular Plant Physiology, Potsdam 14476, Germany; Department of Agricultural, Food and Environmental Sciences, Marche Polytechnic University, via Brecce Bianche, Ancona 60131, Italy

## Abstract

The domestication of crops, coupled with agroecosystem development, is associated with major environmental changes and provides an ideal model of phenotypic plasticity. Here, we examined 32 genotypes of three tetraploid wheat (*Triticum turgidum* L.) subspecies, wild emmer, emmer, and durum wheat, which are representative of the key stages in the domestication of tetraploid wheat. We developed a pipeline that integrates RNA-Seq data and population genomics to assess gene expression plasticity and identify selection signatures under diverse nitrogen availability conditions. Our analysis revealed differing gene expression responses to nitrogen availability across primary (wild emmer to emmer) and secondary (emmer to durum wheat) domestication. Notably, nitrogen triggered the expression of twice as many genes in durum wheat compared to that in emmer and wild emmer. Unique selection signatures were identified at each stage: primary domestication mainly influenced genes related to biotic interactions, whereas secondary domestication affected genes related to amino acid metabolism, in particular lysine. Selection signatures were found in differentially expressed genes (DEGs), notably those associated with nitrogen metabolism, such as the gene encoding glutamate dehydrogenase (GDH). Overall, our study highlights the pivotal role of nitrogen availability in the domestication and adaptive responses of a major food crop, with varying effects across different traits and growth conditions.

## Teaser

Nitrogen had a pivotal role in tetraploid wheat domestication and adaptive responses, leading to diverse impacts on gene expression and selection signatures during primary and secondary domestication.

## Introduction

Domestication influences the genetic diversity of animals and plants as they adapt to agroecosystems and undergo selection to meet human preferences and needs. This process is typically associated with the genome-wide loss of nucleotide diversity due to the combined consequences of selection and genetic drift, which is known as the domestication bottleneck. The loss of genetic diversity has been documented in many domesticated species by comparing them with wild relatives (Bitocchi et al. 2017). A parallel effect is the reprogramming of gene expression and the loss of expression diversity, which was first reported in the common bean (*Phaseolus vulgaris*; Bellucci et al. 2014) and subsequently in other domesticated plants and animals (Sauvage et al. 2017; Liu et al. 2019; Burgarella et al. 2021). Similar observations have been reported at the level of metabolic diversity (Beleggia et al. 2016).

Changes in nucleotide and gene expression diversity during the domestication of tetraploid wheat (*Triticum turgidum* L., 2*n* = 4*x* = 28; AABB genome) are not fully understood. Tetraploid wheat was domesticated in two well-defined phases. Primary domestication from wild emmer (*Triticum turgidum* ssp. *dicoccoides*) to emmer (*Triticum turgidum* ssp. *dicoccum*) started ∼12,000 yrs ago in the Fertile Crescent. This was followed by secondary domestication from emmer to durum wheat (*Triticum turgidum* ssp. *durum*), which started 8,000–10,000 yrs ago in the Near East and gave rise to durum wheat, the most important form of tetraploid wheat and currently the most widespread Mediterranean crop (Gioia et al. 2015; Taranto et al. 2020; Levy and Feldman 2022).

The transition from wild environments to early farming and eventually to modern high-input agroecosystems had profound ecological consequences. Throughout history, humans have employed various methods to enhance soil fertility, such as soil preparation to facilitate organic matter mineralization and the use of livestock manure, with evidence dating back to Neolithic early farming sites ∼7,900 yrs ago (Bogaard et al. 2013). However, the scale and intensity of fertilizer use have escalated over time, especially with the advent of the Haber-Bosch industrial process, which heavily relies on nonrenewable fossil fuels. Notably, the widespread overreliance on nitrogen (N) fertilizers in modern industrial agriculture can be traced back to the Donald model (Donald 1968). This model aims to optimize crop yields by minimizing intraspecific competition and providing substantial agronomic inputs, including fertilizers (Fréville et al. 2022). Today, the global application of N fertilizers, particularly to cereal crops, exceeds 80 million tons annually (Ludemann et al. 2022). N is an essential macronutrient whose availability is directly linked to crop yield and grain quality (protein content) (Barneix 2007; Howarth et al. 2008; Laidò et al. 2013), but it is also directly harmful to humans and the environment. Indeed, excess N from agricultural sources is one of the major fresh water pollutants, causing the eutrophication of aquatic ecosystems (Rockström et al. 2009). The production of industrial fertilizers contributes ∼3% of global CO_2_ and is a primary source of N_2_O (Wood and Cowie 2004). Understanding genetic variations in N acquisition, assimilation, and metabolism can therefore provide strategies for crop improvement to meet the United Nations Sustainable Development Goals (SDGs) (Plett et al. 2018; Hawkesford and Griffiths 2019).

Environmental changes that accompanied the domestication of crops over thousands of years can be tolerated by organisms that exhibit phenotypic plasticity, defined as the ability of a genotype to exhibit changes in a specific trait across different environments, and through the modulation of gene expression (Bradshaw 1965; Laitinen and Nikoloski 2019). Understanding the molecular basis of phenotypic plasticity in crops and their wild relatives can help to address the challenges faced by modern agriculture. In tetraploid wheat, phenotypic differences in below-ground and above-ground growth traits related to N availability primarily arose during secondary domestication (Gioia et al. 2015), but the relationship between N metabolism and changes in gene expression plasticity during domestication is unclear.

Here, we analyzed 32 wild emmer, emmer, and durum wheat genotypes by RNA-Seq in contrasting N availability scenarios, to investigate the potential role of N during the domestication of tetraploid wheat. Our study elucidates the subspecies-specific responses to nitrogen, as well as nucleotide and gene expression diversity during both primary and secondary domestication phases. Our results provide insight into the pivotal role of N during the domestication and adaptive plasticity of one of our major food crops.

## Results and discussion

### Choice of the reference genome: mapping accuracy and comparative analysis across available tetraploid wheat references

The inclusion of genotypes from three distinct tetraploid wheat subspecies (i.e. wild emmer, emmer, and durum wheat) within our panel presented challenges when comparing results across groups. In this context, the choice of the reference genome was of crucial importance. Given that reference genomes were only available for two of the three subspecies examined in our study, namely wild emmer and durum wheat, we considered using the A and B subgenomes of bread wheat (*Triticum aestivum*) as an outgroup reference closely related to all subspecies in the panel. This option aimed to mitigate potential biases that might arise from favoring any subspecies, ensuring a balanced comparison across the genotypes. Indeed, the A and B subgenomes of bread wheat serve as a good intermediate between emmer (wild and domesticated) and durum wheat. Bread wheat shares ancestral A and B subgenomes with the wild and cultivated emmer, originating from the same founding population. On the other hand, the A and B subgenomes of durum wheat underwent differentiation coinciding with the origin of bread wheat (Haudry et al. 2007; Levy and Feldman 2022).

However, we rigorously tested these assumptions and validated our choice by also mapping reads to currently available tetraploid wheat reference genomes: wild emmer Zavitan (Zhu et al. 2019) and durum wheat Svevo (Maccaferri et al. 2019).

We prepared 128 RNA-Seq libraries from the 4-week-old leaves of 32 tetraploid wheat genotypes representing wild emmer, emmer, and durum wheat, grown in two contrasting N conditions (N starvation and optimal N availability) (Supplementary Data Set 1). The mapping frequency across the entire genome was consistent among the three references (86% to 87%) with an average of 6.8 million mapped reads per genotype (Supplementary Data Set 1A to C). However, while the proportion of reads mapping to genic regions was similar when comparing bread wheat and wild emmer wheat (average 73%), it was lower when using the durum wheat reference genome (average 52%) primarily due to the absence of untranslated regions (UTRs) in the Svevo reference annotation (Supplementary Data Set 1A to C).

Pairwise genetic distances between the three reference genomes were computed using an alignment-free method based on the MinHash technique (Ondov et al. 2016). This method compresses large genomic sequences (the 3 entire genomes, in our case) into sketch representations, allowing for rapid similarity estimations with bounded error. We found that the Mash distance (*D*), which is an approximation of the mutation rate (Ondov et al. 2016), between the bread wheat Chinese Spring A and B subgenomes was ∼0.014 (*P-*value < 10^−10^) when compared with wild emmer Zavitan, and ∼0.008 (*P-*value < 10^−10^) when compared with durum wheat Svevo. The distance between durum wheat Svevo and wild emmer Zavitan was the same as that between bread wheat and wild emmer Zavitan (*D* ∼0.014, *P-*value < 10^−10^). From the resulting distances, the average nucleotide identity (ANI) can be extracted as *D* ∼1—ANI (Ondov et al. 2016), obtaining a value for ANI of ∼99% between each genome, thus confirming their close relationship.

Computing raw read counts and filtering out genes with weak expression (see Materials and Methods) resulted in 32,358 genes from the bread wheat Chinese Spring reference genome. We performed the same process using the wild emmer Zavitan and durum wheat Svevo reference genomes, resulting in 33,586 and 29,784 genes, respectively. A comprehensive comparison of the sequences of the three gene sets also showed an ANI exceeding 98%.

Overall, despite minor differences in the outcomes across different references, the overall patterns remain consistent, and the use of an outgroup species for reference might help mitigate biases and ensure fair representation of all subspecies, maintaining mapping accuracy and coverage of gene regions, making it a suitable choice for our analysis.

### A greater loss of nucleotide diversity occurred during the secondary domestication of tetraploid wheat

Variant calling on the RNA-Seq of the whole panel of 32 genotypes produced 800,996 high-quality single-nucleotide polymorphisms (SNPs) including “population-specific” SNPs found only in one of the three subspecies. The number of polymorphic sites was similar in wild emmer (617,128) and emmer (613,509), but was much lower in durum wheat (425,513). Site frequency spectra for each subpopulation are provided in Supplementary Fig. S1. SNPs were categorized as “private” if found exclusively in a single subspecies or “shared” if they were distributed across two or three subspecies. We identified 190,377 common SNPs shared by all three *taxa*. As expected, wild emmer and emmer shared the highest percentage of SNPs (33%, 206,578). In contrast, durum wheat shared only 11% (46,352) of its SNPs with wild emmer and 17% (71,147) with emmer (Supplementary Data Set 2).

**Figure 1.**
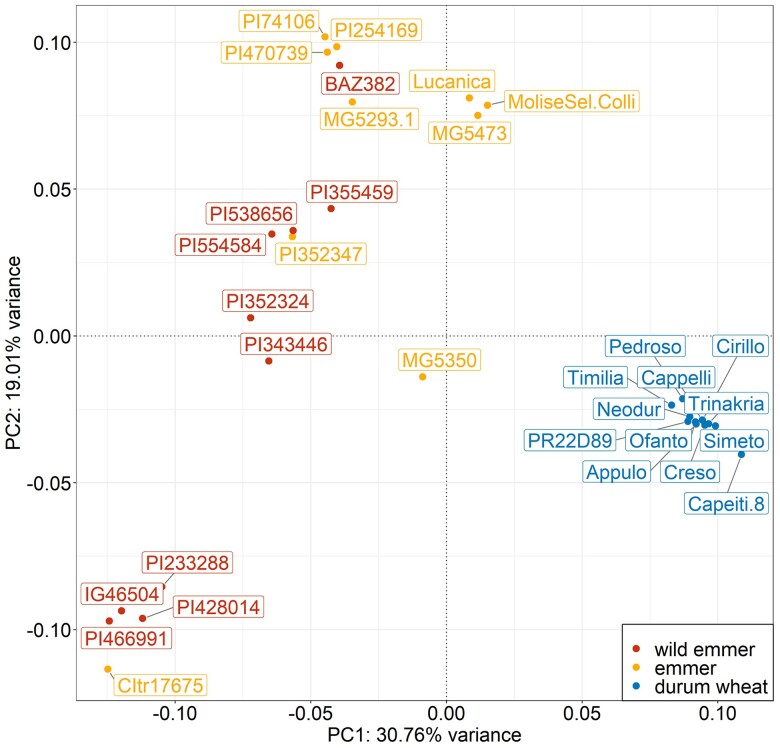
Principal component analysis (PCA) of 32 wheat genotypes based on single-nucleotide polymorphisms (SNPs). The first two principal components (PC1 and PC2) are shown. The three colors represent different *taxa*. Labels show the accession name of each genotype.

SNP principal component analysis (PCA) revealed the broad genetic structure of the three wheat *taxa* (Fig. 1) and confirmed that secondary domestication had a greater impact than primary domestication in differentiating the durum wheat subspecies. The 12 durum wheat genotypes are genetically very similar, forming a dense cluster that is clearly distinguishable from the wild emmer and emmer genotypes. In contrast, the wild emmer and emmer genotypes are loosely clustered, indicating a greater genetic admixture. These results are consistent with previous genetic studies on the origins of domesticated tetraploid wheat and reflect the multiple stages of domestication (Haudry et al. 2007; Luo et al. 2007; Civáň et al. 2013; Oliveira et al. 2020).

Nucleotide diversity estimates (*π* and *θ*) revealed the expected substantial loss of diversity during domestication, highlighting the greater impact of secondary domestication (Table 1). Considering *π*, the average nucleotide diversity of durum wheat was ∼17% lower than that of domesticated emmer, which in turn was ∼11% lower than that of wild emmer. The cumulative effect of primary and secondary domestication was a ∼26% reduction in the nucleotide diversity of durum wheat compared to its wild ancestor (Table 1).

**Table 1.** Nucleotide diversity estimates and diversity loss for the three wheat *taxa*

	Wild emmer	Emmer	Durum wheat	Loss of nucleotide diversity (%)		
				Lpd	Lsd	Both
*Π*	0.0050	0.0045	0.0037	11.4	16.8	26.3
*θ*	0.0047	0.0040	0.0029	15.3	27.2	38.3

Diversity loss is shown during primary domestication (wild emmer to emmer, Lpd), secondary domestication (emmer to durum wheat, Lsd), and both processes (wild emmer to durum wheat), based on average *π* and *θ* estimates of nucleotide diversity.

To ensure that our results were not biased toward the chosen reference genome, we also repeated the variant calling, PCA based on SNPs, and nucleotide diversity estimates for each subspecies using the wild emmer and durum wheat reference genomes to allow comparison with the bread wheat reference. The numbers of SNPs, nucleotide diversity estimates, and diversity loss estimates are summarized in Supplementary Data Set 2. Both the wild emmer and durum wheat references yielded fewer SNPs (604,479 and 544,406, respectively) compared to using the bread wheat reference (800,996). However, the similarity in the number of polymorphic sites between wild emmer and emmer along with the lower number of durum wheat SNPs, as well as the ratio between private and shared SNPs, was reaffirmed with both of these alternative references (Supplementary Data Set 2). The equivalence in the utilization of the three references was reinforced by identical PCA results obtained from the two sets of SNPs derived from the calls using wild emmer and durum wheat references (Supplementary Fig. S2).

In addition, the estimates of *π* and *θ* for wild emmer and emmer exhibited lower subspecies-specific values compared to the bread wheat reference (Supplementary Data Set 2). Considering *π*, the values obtained using the wild emmer reference were 0.0027 for wild emmer, 0.0027 for emmer, and 0.0024 for durum, whereas similar but lower values were obtained when using the durum wheat reference, especially for durum wheat (*π* = 0.0011). Even so, the overall trend of diversity loss confirmed previous findings using the bread wheat reference, indicating a more pronounced impact of secondary domestication. The percentage losses of *π* nucleotide diversity observed using the bread wheat reference (11.4 for primary domestication and 16.8 for secondary domestication) were in the range obtained using wild emmer (3.2 for primary domestication and 10.1 for secondary domestication) and durum wheat (12.5 for primary domestication and 48.1 for secondary domestication).

In summary, although we observed slight variations in SNP detection and nucleotide diversity estimates across various reference genomes, the general trends of genetic variation and diversity loss remained consistent. By detailing our methodology for testing available reference genomes, we aim to provide guidance for other researchers encountering similar challenges. In the case of tetraploid wheat, we propose that the use of an outgroup species as a reference genome does not introduce bias in gene identification and subsequent analyses. On the contrary, it may mitigate biases and ensure fair representation for all subspecies in the study.

### The variability of gene expression during domestication was influenced by N availability

**Figure 2.**
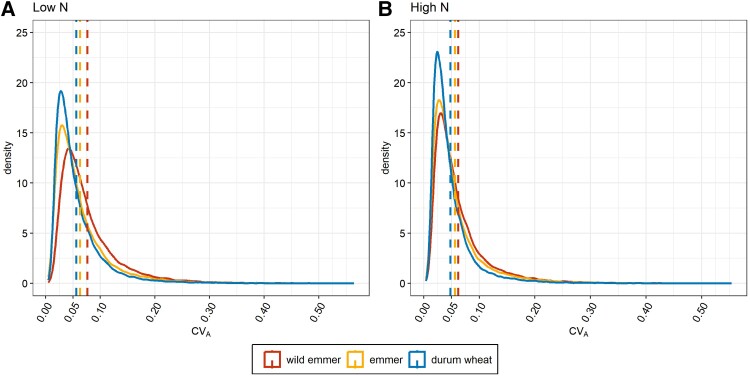
Density plots of the additive coefficient of variation (CV_A_) in the three wheat *taxa*. Comparison of the estimated density functions of the CV_A_ in gene expression, calculated using all 32,358 genes. **A)** Low-N conditions. **B)** High-N conditions. Dashed lines represent the averaged CV_A_ value, colored according to the different *taxa*.

To quantify the diversity of gene expression in each subspecies, we calculated evolvability scores under high- and low-N availability conditions. Evolvability was estimated using the additive coefficient of variation (CV_A_) in read counts (Supplementary Data Set 3). In contrast to heritability, CV_A_ is a standardized measure of additive genetic variation that is not influenced by other sources of variance (Houle 1992; Hansen et al. 2011) and is therefore well suited for comparative analysis (Garcia-Gonzalez et al. 2012; Gioia et al. 2015). We found that the CV_A_ was the highest in wild emmer, followed by emmer and then durum wheat, indicating a decline during domestication under both N availability conditions (Fig. 2A and B; Table 2). The loss of diversity in gene expression has been observed across the domestication process in other crops, such as common bean (Bellucci et al. 2014), tomato (*Solanum lycopersicum*) (Sauvage et al. 2017), and sorghum (Sorghum bicolor) (Burgarella et al. 2021) as well as domesticated animal species (Liu et al. 2019). However, we observed a higher mean CV_A_ across all three subspecies under low-N compared to high-N conditions (Fig. 2A and B; Table 2). This suggests that higher N availability promotes a more uniform gene expression pattern, whereas higher variability (plasticity) is observed during N starvation.

**Table 2.** Mean additive coefficient of variation (CV_A_) in gene expression and loss of expression diversity for the three wheat *taxa*

	Wild emmer	Emmer	Durum wheat	Loss of expression diversity (%)		
				Lpd	Lsd	Both
CV_A_ high N	0.062	0.056	0.048	9.1	14.5*	22.3
CV_A_ low N	0.076	0.063	0.056	17.6*	11.1*	26.7

Diversity loss is shown during primary domestication (wild emmer to emmer, Lpd), secondary domestication (emmer to durum wheat, Lsd), and both processes (wild emmer to durum wheat), based on averaged CV_A_ values calculated for all 32,358 genes.

^*^ *P* < 0.001, Mann–Whitney *U*-test for difference between Lpd and Lsd within each N condition and difference between high N and low N within Lpd and within Lsd.

We used the contrasting N conditions of our samples to examine whether the loss of expression diversity is associated with the specific aspects of the cultivation environment, causing primary and secondary domestication to have a substantially different impact. Under high-N conditions, we observed a ∼9% loss in expression diversity in emmer compared to wild emmer (effect of primary domestication) and a ∼15% loss in durum wheat compared to emmer (effect of secondary domestication). In contrast, these losses were ∼18% and 11% under N starvation conditions, revealing twice the loss of expression diversity during primary domestication, but a lower value during secondary domestication (Table 2). All four values differed significantly from each other (Mann–Whitney *U*-test, *P* < 0.001). The opposing expression diversity profiles during domestication under high-N and low-N conditions were observed not only for overall gene expression but also for the subgroup comprising all differentially expressed genes (DEGs) and the subgroup comprising all unmodulated genes (Supplementary Table S1). The loss of expression diversity among the DEGs due to primary domestication was ∼9% and ∼15% under high-N and low-N conditions, respectively, whereas the corresponding losses due to secondary domestication were ∼18% and ∼14% (Supplementary Table S1). The loss of expression diversity among the unmodulated genes was similar to the values for overall gene expression (Supplementary Table S1).

A phenotypic study of the same accessions used in the present work has already shown that secondary domestication reduced the phenotypic diversity under high-N conditions, but the reduction was smaller and not significant under N starvation (Gioia et al. 2015). In the case of durum wheat, selection has apparently enhanced the growth response to N availability, indicating a putative focus on improving N uptake and utilization efficiency. Our expression diversity results indicate that selection may have favored specific traits and thus led to a more uniform set of cultivars, as also suggested in an earlier study based on morphological traits (Gioia et al. 2015).

### Domestication and nitrogen availability played a role in the divergence of tetraploid wheat

**Figure 3.**
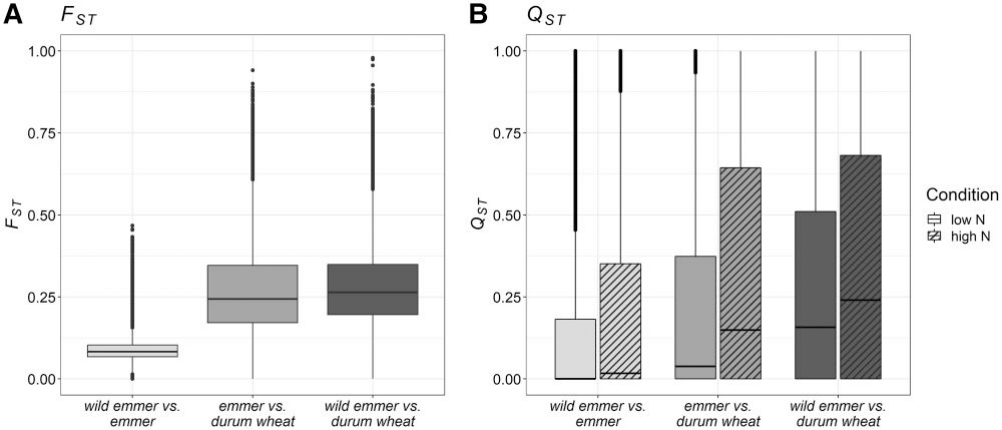
*F*_ST_ and *Q*_ST_ distributions. **A)** Boxplots showing the gene locus *F*_ST_ distribution for every subspecies pairwise comparison. **B)** Boxplots showing the gene expression *Q*_ST_ distribution for every subspecies pairwise comparison under low-N and high-N conditions, represented by empty and hatched grayscale bars, respectively. The borders of the box represent the 25th and 75th percentiles. The horizontal line in the middle of the box represents the median. Whiskers extend to the minimum and maximum values, unless a point exceeds 1.5 times the interquartile range, in which case the whisker extends to this value and values beyond are plotted as individual points (outliers).

Genetic differentiation among the three subspecies was estimated by calculating the pairwise fixation index (*F*_ST_) for every gene locus in our dataset. As shown in Fig. 3A, the lowest genetic differentiation was observed between wild emmer and emmer (median *F*_ST_, ∼0.08), whereas much higher genetic differentiation was found between emmer and durum wheat (median *F*_ST_, ∼0.24) and, similarly, between wild emmer and durum wheat (median *F*_ST_, ∼0.26). These values align with earlier findings that examined broad collections of tetraploid wheat accessions (Luo et al. 2007), suggesting an indication of the representativeness of the utilized genotypes.

Divergence at the transcriptomic level was estimated by calculating *Q*_ST_, the quantitative analog of *F*_ST_, taking N availability into account as an environmental variable. Under both N conditions, we observed the same trend shown for *F*_ST_ (Fig. 3B). Specifically, secondary domestication had a stronger impact on differentiation (emmer *vs* durum wheat; median *Q*_ST LN_, ∼0.04; median *Q*_ST HN_, ∼0.15) than primary domestication (wild emmer *vs* emmer, median *Q*_ST LN,_ ∼4.7 × 10^−9^, median *Q*_ST HN_ ∼0.018). Interestingly, the *Q*_ST_ distributions of every pairwise comparison showed higher values under high-N conditions compared to N starvation (Fig. 3B), suggesting that the response to N availability has been under selection during domestication and breeding substantially contributed to the differentiation of gene expression in tetraploid wheat in response to different agroecosystems.

We therefore dissected the transcriptome as a multidimensional plastic phenotype, in which the abundance and expression patterns of thousands of genes in different environmental conditions can be processed as phenotypic traits, reflecting variability in levels of gene expression. Studying the transcriptome as a plastic phenotype is a powerful approach to understand the molecular basis of plasticity and its evolutionary potential (Leinonen et al. 2013; Oostra et al. 2018). By assessing how gene expression patterns over an environmental gradient vary within populations (reflecting phenotypic plasticity) and among populations (reflecting genetic differentiation), it is possible to identify candidate genes and regulatory pathways that may be under selection for adaptation (Oleksiak et al. 2002; Whitehead and Crawford 2006a, 2006b). In tetraploid wheat, the *Q*_ST_*–F*_ST_ comparison method has been used to detect selection signatures for metabolites, treated as molecular phenotypic traits (Beleggia et al. 2016). Until now, despite its suitability for the analysis of gene expression data, this method has been rarely adopted in transcriptomics studies (Roberge et al. 2007; Kohn et al. 2008; Aykanat et al. 2011).

We implemented a methodology based on *Q*_ST_ distributions and *Q*_ST_*–F*_ST_ comparisons to perform a “selection scan,” seeking genes whose expression was potentially under selection. To ensure the reliability of gene selection and minimize the risk of false positives, we established two thresholds based on the distributions of gene expression heritability (Supplementary Fig. S3), while acknowledging that we developed this methodology as a proof of concept with a limited number of genotypes. Consequently, we aimed to establish a high confidence level to ensure the robustness of our approach. Genes with *H*^2^ < 0.7 were removed in order to retain only candidates in the top 15% of the distribution for which we can ascertain that their variation is predominantly genetic. However, to consider those genes whose expression was strongly influenced by N availability, we also evaluated the percentage of the species × environment (*S × N*) variance component (i.e. every species subgroup × N condition), retaining those genes meeting at least the 20% threshold (Supplementary Fig. S3). Altogether, 5,868 genes (∼18% of the total number) met these criteria and only those with *Q*_ST_ values in the 5% right-hand tail of the distributions were considered candidates for selection. The *Q*_ST_ and *F*_ST_ values of the filtered genes were then compared (Supplementary Fig. S4) to confirm that their divergent expression (high *Q*_ST_ values) was caused by directional selection (*Q*_ST_ > *F*_ST_) and not by genetic drift (*Q*_ST_ ≈ *F*_ST_) or stabilizing selection (*Q*_ST_ < *F*_ST_) (Bonnin et al. 1996; Whitlock and Guillaume 2009). After removing *F*_ST_ values < 0.01, we observed that all the resulting 967 genes satisfied the criterion *Q*_ST_ > *F*_ST_, indicating that their expression was probably subjected to directional selection in at least one of the evolutionary contexts examined herein (i.e. primary and/or secondary domestication under high-N and/or low-N availability conditions). Notably, ∼280 genes were consistently detected in each of the six comparisons (Supplementary Data Set 4). These genes exhibited high *Q*_ST_ values, indicating substantial divergence in expression levels, coupled with lower *F*_ST_ values, suggesting limited nucleotide-level divergence. This suggests that the observed signals likely arise from upstream regulatory mechanisms influencing gene expression, rather than mutations within the gene's coding regions, potentially leading to altered gene products. Consequently, we conducted SNP annotation on the 967 genes under selection, accounting for both synonymous and non-synonymous mutations. We then compared these results by annotating variants present in another set of 967 genes randomly selected from the total pool of 32,358 genes. As detailed in Supplementary Table S2, for each *taxon*, we detected an average lower number of both synonymous and non-synonymous mutations in the genes under selection compared to the randomly selected group. Specifically, the ratios of non-synonymous/synonymous mutations were ∼0.78 for selected genes and ∼0.81 for random ones in wild emmer and emmer wheat, showing comparable values. However, in durum wheat, this ratio was significantly lower: ∼0.69 for selected genes and ∼0.76 for random ones (Kolmogorov–Smirnov two-sided test *P* < 2.2e-16). These findings bolster our identified signals of selection, suggesting that our 967 candidate genes, precisely because they are putatively under directional selection, may have also undergone a process of purifying selection, thereby preventing the accumulation of deleterious mutations.

Gene Ontology (GO) enrichment analysis was performed on the six groups of genes. Not all six comparisons yielded significant results, but significant and distinct GO categories emerged specifically during primary and secondary domestication under high-N (Supplementary Fig. S5). However, examining the entire evolutionary process (from wild emmer to durum wheat), categories associated with the amino acid biosynthesis were enriched also in genes showing selection signatures when comparing wild emmer and durum wheat under low-N conditions (Supplementary Fig. S5). Our results suggest that primary and secondary domestication involved different natural and artificial selection pressures affecting distinct sets of genes and phenotypes influenced by N availability, indicating that certain genetic pathways may have been particularly important for the adaptation of wheat to different environmental conditions during its domestication history.

Among the genes under selection during primary domestication, categories linked to “defense-related programed cell death, modulated by biotic interactions” were enriched, suggesting an enhanced hypersensitive response to pathogens. As wild genotypes transition to agroecosystems characterized by dense crop monocultures, they encounter increased disease pressure from crop-specific pathogens (Savary et al. 2019). This prompts a hypersensitive response, potentially leading to programmed cell death and necrosis as a defense mechanism. Pathogen defense mechanisms in plants often intersect with the regulation of beneficial symbiotic interactions, suggesting a tradeoff between symbiosis-associated traits and innate immunity (Porter and Sachs 2020). Moreover, domesticated crops are less able to leverage microbial interactions compared to wild counterparts, as shown by comparative studies involving bread wheat landraces and old *vs* modern varieties (Valente et al. 2023). This reduced capacity may in part reflect the widespread adoption of high-input agricultural practices, wherein the availability of fertilizers diminishes the need for plants to invest in symbiotic relationships (Martín-Robles et al. 2018).

Among the genes under selection during secondary domestication, we observed the enrichment of categories associated with amino acid metabolism, particularly those related to the “lysine catabolic process” (Supplementary Fig. S5). This included genes encoding the bifunctional enzyme lysine ketoglutarate reductase/saccharopine dehydrogenase (LKR/SDH), which breaks down lysine via the saccharopine pathway (SACPATH). The structure and transcription of the LKR/SDH gene have been investigated in durum wheat, revealing species-dependent differences in expression and lineage-specific variations between monocots and dicots (Anderson et al. 2010). Lysine is a limiting essential amino acid in cereal grains, and efforts have been made to enhance its content in crops like maize (*Zea mays*) and rice (*Oryza sativa*) by targeting the catabolic pathway (Houmard et al. 2007; Frizzi et al. 2008; Long et al. 2013). However, lysine-rich proteins generally do not accumulate to high levels in cereal seeds, which instead stockpile prolamins (such as gliadin in wheat). The SACPATH appears to direct lysine toward the production of glutamic acid, a precursor of proline, which is abundant in gluten (Arruda and Barreto 2020).

Evolutionary metabolomics has revealed signatures of selection affecting amino acid metabolism during secondary domestication (Beleggia et al. 2016). Changes in amino acid metabolism during domestication have been observed in crops such as sunflower, maize, and common bean based on nucleotide data (Chapman et al. 2008; Swanson-Wagner et al. 2012; Bellucci et al. 2014). In durum wheat, domestication has been associated with the selection of specific protein compositions, reducing the diversity of gliadin and glutenin subunits, thus affecting grain yield and gluten properties (Laidò et al. 2013, 2014). Moreover, the SACPATH is upregulated in response to drought stress in spring wheat genotypes, particularly in drought-tolerant varieties, suggesting a role in stress adaptation (Michaletti et al. 2018). Proline, derived from this pathway, may serve as a major constituent of storage proteins and also a key osmoprotectant produced in response to stress (Kavi Kishor et al. 2022). Overall, selection during wheat domestication may have influenced the expression of SACPATH genes, favoring not only protein composition but also abiotic stress tolerance.

### Changes in nitrogen availability trigger gene expression, resulting in a twofold increase in the number of differentially expressed genes in durum wheat compared to emmer and wild emmer wheat

**Figure 4.**
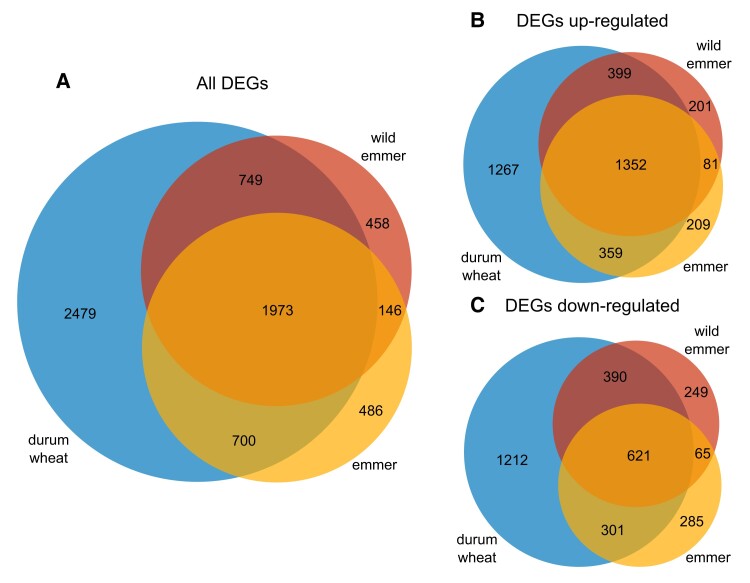
Venn diagrams showing differentially expressed genes (DEGs) when comparing high-N and low-N conditions within each subspecies. **A)** Total set of DEGs, **B)** upregulated DEGs only, and **C)** downregulated DEGs only.

We identified DEGs in each subspecies that discriminated between high-N conditions and N starvation using a stringent pipeline and strict thresholds (*P*_adj_ < 0.001) to reduce the number of false positives. We found 3,326 DEGs in wild emmer, 3,305 in emmer and 5,901 in durum wheat, with more upregulated than downregulated genes in all three subspecies. Durum wheat had the highest percentage of private DEGs (∼42%, 2,479), whereas similar numbers were found in wild emmer (∼14%, 458) and emmer (∼15%, 486). Wild emmer and emmer shared ∼23% (749) and ∼21% (700), respectively, of their DEGs with durum wheat. The percentage of DEGs shared only between wild emmer and emmer was 4% (146), but almost 60% of wild emmer and emmer DEGs and ∼33% of durum wheat DEGs were shared by all three *taxa* (Fig. 4A). The proportions of private and shared DEGs were preserved when we separated them into upregulated and downregulated subsets (Fig. 4B and C). In all three *taxa*, most DEGs were located on chromosomes 2A, 2B, 3A, 3B, 5A, and 5B, each carrying >7.5% of the DEGs, whereas chromosomes 6A and 6B each contained only ∼5% of the DEGs (Supplementary Fig. S6).

GO enrichment analysis of the DEGs meeting the threshold FDR < 0.05 revealed 23 macro-categories in wild emmer, 21 in emmer, and 25 in durum wheat (Supplementary Fig. S7). The main differences between the three subspecies were observed for categories related to “signaling,” “regulation of biological process,” “developmental process,” and “metabolic process” (Supplementary Fig. S7). We observed the uniform enrichment of GO categories associated with upregulated genes in all three subspecies, including terms linked to N and amino acid metabolism as well as carbon (C) metabolism and photosynthesis (Supplementary Fig. S7). In contrast, the enrichment of GO categories associated with downregulated genes was more selective, with some GO categories related to N metabolism enriched only in durum wheat, including GO:0006807 and GO:0034641 (N compound and cellular N compound metabolic process, respectively) and GO:0006536 “glutamate metabolic process” (Supplementary Data Set 5). Functional annotations of the most strongly modulated genes (top 5% |log_2_FC| values) are reported in Supplementary Data Set 6.

Our data confirm, on a larger set of samples, earlier observations on the response of wheat to N starvation based on transcriptomics and metabolomics data. These earlier studies included one emmer and one durum wheat genotype also present in our sample set (Beleggia et al. 2021), but also considered the durum wheat cultivar Svevo (Curci et al. 2017) and various bread wheat cultivars (Sultana et al. 2020). As expected, genes involved in N metabolism were modulated during N starvation. Among the key genes for N assimilation, those encoding asparagine synthetase and nitrite reductase were upregulated in every *taxon*, whereas those encoding glutamate carboxypeptidase and glutamate decarboxylase were downregulated. We observed contrasting profiles for genes encoding ureide permease (a ureide transporter), which were strongly upregulated in all three subspecies in response to N stress, whereas genes encoding nitrate transporters were strongly downregulated. The modulated genes also included transporters of amino acids and other nutrients.

N starvation also influenced other metabolic pathways, revealing many further DEGs involved in C metabolism, especially fatty acid metabolism, glycolysis, photosynthesis, and the tricarboxylic acid (TCA) cycle. About 10% of the highest-ranking DEGs represented transcription factors and protein kinases. The most common functional category (accounting for 17% of annotated DEGs) reflected the general stress response to N starvation, including the mitigation of oxidative stress and detoxification. Examples included genes encoding cytochrome P450s, glutaredoxin family proteins, glutathione S-transferases, and peroxidases (Supplementary Data Set 6).

To compare gene expression between the three *taxa* while taking the environmental effects into account, we also identified DEGs between each pair of subspecies under all N conditions. Accordingly, we compared emmer *vs* wild emmer (primary domestication, high- and low-N), durum wheat *vs* emmer (secondary domestication, high- and low-N), and durum wheat *vs* wild emmer (cumulative effect, high- and low-N) (Supplementary Fig. S8). The wild emmer *vs* emmer comparison revealed few DEGs regardless of N availability (12 and 11 DEGs under high-N and low-N conditions, respectively), whereas the emmer *vs* durum wheat comparison revealed 41 DEGs associated with high N and 29 associated with N starvation, and the wild emmer *vs* durum wheat comparison revealed 46 DEGs associated with high-N and only 10 associated with N starvation. These data indicate that the number of DEGs increases during domestication but only when there is a sufficient N supply (Supplementary Fig. S8). Interestingly, there were more upregulated than downregulated genes in all pairwise comparisons under high-N conditions (∼65%) but the proportion increased under N starvation, particularly for the comparison of wild emmer *vs* durum wheat (90%). The preponderance of upregulated genes during domestication has also been observed in maize (Lemmon et al. 2014), whereas domestication was shown to increase the proportion of downregulated genes in common bean (Bellucci et al. 2014), eggplant (*Solanum melongena*) (Page et al. 2019), and sorghum (Burgarella et al. 2021) landraces compared to wild relatives. The absence of consistent patterns suggests that the evolution of domesticated phenotypes is driven by specific processes that are unique to each crop.

Among the 102 DEGs found in at least one of the six pairwise comparisons between subspecies (Supplementary Data Set 7), 35 were also found among DEGs identified between contrasting N conditions and 24 of these were proposed to be under selection. Overall, six genes were identified in all three experiments (i.e. differentially expressed between subspecies and between contrasting N conditions, and showed evidence of selection).

### Selection shaped the expression profiles of genes modulated by nitrogen availability

**Figure 5.**
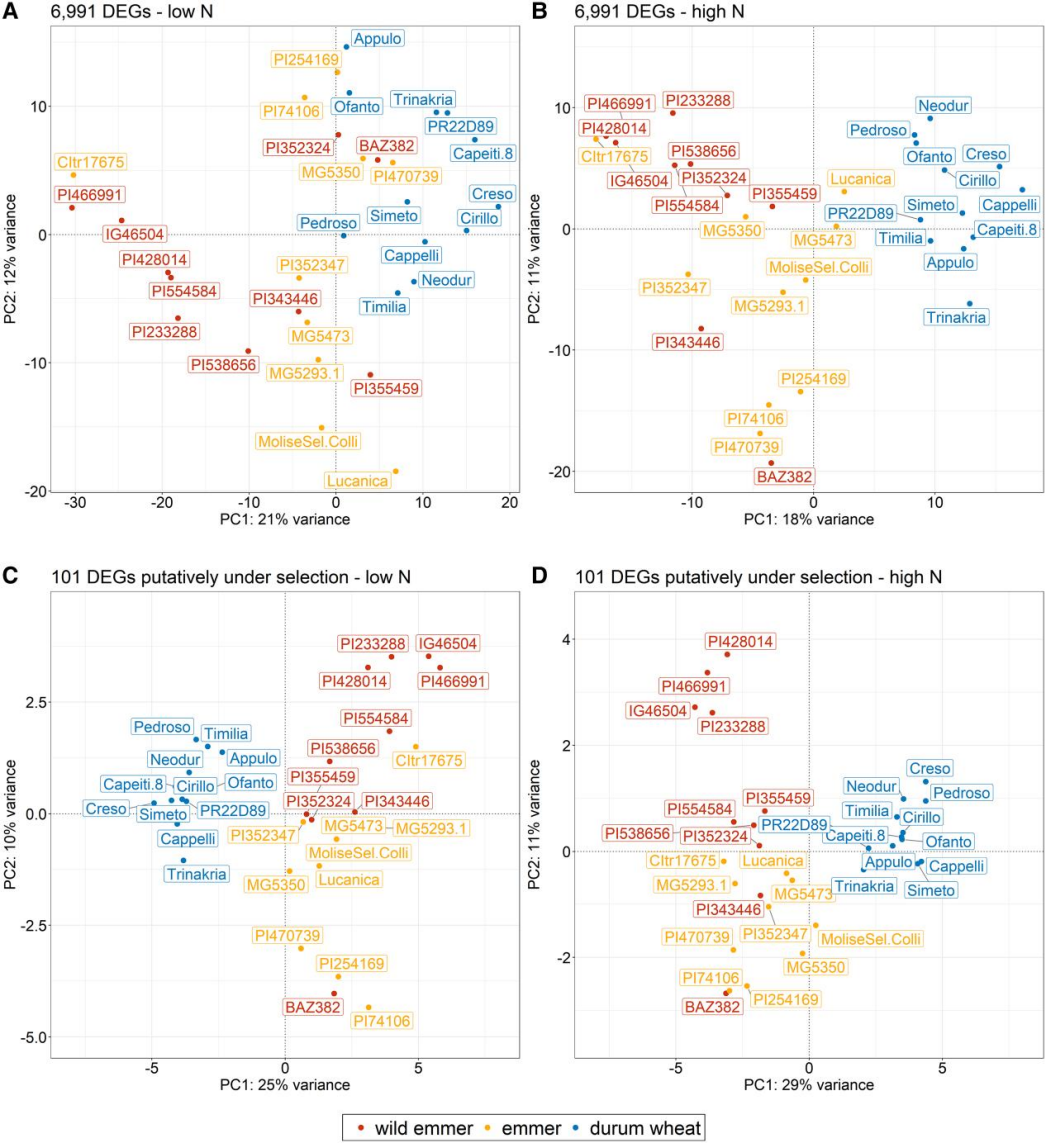
Principal component analysis (PCA) of 32 wheat genotypes based on expression data of differentially expressed genes (DEGs) when comparing high-N and low-N conditions within each subspecies. **A, B)** Plots based on all 6,991 DEGs (not filtered): A low-N conditions and B high-N conditions. **C, D)** Plots based on 101 DEGs that are also putatively under selection: C low-N conditions and D high-N conditions. Samples are represented by *taxon*-specific colored dots. Labels show the accession name of each genotype.

The 6,991 DEGs found in at least one species when comparing contrasting N conditions included 101 putatively under selection, which are candidates for the adaptive response to N availability. The expression profiles of these selected genes, in all three subspecies under both N conditions, are shown in Supplementary Fig. S9. We applied PCA to the normalized read counts in order to determine whether the different genotype groups can be separated based on their gene expression. Initially, we incorporated all 6,991 DEGs (Fig. 5A and B) before focusing on the subset of 101 DEGs that were also putatively under selection (Fig. 5C and D). When considering all DEGs, PC1 did not completely separate the durum wheat genotypes from the other *taxa*, in contrast to the clear separation observed for the SNP data (Fig. 1), and this was particularly evident during N starvation (Fig. 5A). There was also a moderate degree of overlap between the wild emmer and emmer genotypes along PC2. However, when we focused on the DEGs under selection, PC1 separated the durum wheat genotypes into a densely clustered group (as observed for the SNP data) under both N conditions, and PC2 separated the wild emmer and emmer genotypes more clearly, especially under high-N conditions (Fig. 5C and D). For an overview of overall gene expression patterns in the two N conditions, please refer to Supplementary Fig. S10, which illustrates the PCA conducted on the complete set of genes (32,358).

The selection signatures (based on *Q*_ST_–*F*_ST_ values) allowed us to identify genes that have putatively diverged between tetraploid wheat subspecies due to selection pressure. By integrating this information with classical differential expression analysis, which identified genes that are differentially expressed under contrasting N conditions, it was possible to pinpoint 101 candidate genes that are both genetically differentiated and functionally relevant to the environmental factor of interest: N availability during the domestication and diversification of cultivated wheat. Functional annotation (Supplementary Data Set 8) revealed upregulated genes associated with C metabolism as well as some encoding transcription factors and transporters, as well as both upregulated and downregulated genes associated with general stress responses and N metabolism, specifically those encoding enzymes involved in amino acid metabolism such as methionine aminopeptidase, aspartokinase and glutamate dehydrogenase (GDH). The latter is particularly noteworthy because, in addition to its modulation in response to different N conditions and the presence of selection signatures, the *GDH* gene was also upregulated in the comparison between wild emmer and durum wheat under high-N conditions (Supplementary Data Set 7). GDH is a key enzyme involved in N metabolism and N/C balance (Miflin and Habash 2002). This is supported by the co-localization of quantitative trait loci for GDH activity and physiological traits associated with the flag leaf lamina, such as soluble protein and amino acid content, as well as flag leaf area and dry weight (Fontaine et al. 2009). Selection signatures were also identified in the *GDH* gene when comparing landraces with old and modern durum wheat cultivars (Taranto et al. 2020). Our results confirm that N metabolism has been a key driver during the evolutionary history of wheat, particularly the central role of glutamate in the process of domestication. This was also suggested by a combined transcriptomics and metabolomics study showing that glutamate and γ‐aminobutyric acid (mainly synthesized from glutamate) are central to the genotype-specific response of emmer and durum wheat to N starvation (Beleggia et al. 2021).

We have shown that significant changes occurred at the nucleotide and gene expression levels during the domestication of tetraploid wheat, taking into account the environmental variable of N availability. We observed that more nucleotide diversity has been lost during secondary domestication compared to primary domestication. In addition, we found a parallel trend in the loss of gene expression diversity associated with the domestication process, with a stronger effect due to secondary domestication. The observed loss of expression diversity may be related to N availability in the durum wheat selection environment. Our findings suggest that selection may have operated in different directions during primary and secondary domestication, the former involving changes related to biotic interactions and the latter related to amino acid metabolism.

Despite the limited number of genotypes available for our study, the innovative combination of RNA-Seq analysis and the estimation of quantitative genetic parameters allowed us to develop a pipeline for the identification of selection signatures and phenotypic plasticity in gene expression data based on evolvability and *Q*_ST_–*F*_ST_ scores. Emphasizing the pioneering nature of our work, we opted to introduce stringent and high confidence thresholds for the considered parameters, aiming to present our methodology as a proof of concept. While presenting promising results, we acknowledge the potential for further refinement and adjustment of the methodology in experiments employing larger genotype datasets. The set of genes we identified with underlying selection signatures will facilitate the development of innovative strategies to improve resource use efficiency and environmental sustainability in crop management.

## Materials and methods

### Plant material and experimental design

The study included 32 tetraploid wheat genotypes, comprising 10 accessions of wild emmer (*T. turgidum* ssp. *dicoccoides*), 10 accessions of emmer (*T. turgidum* ssp. *dicoccum*), and 12 accessions of durum wheat (*T. turgidum* ssp. *durum*) (Supplementary Data Set 1). The samples analyzed in our study were selected from a larger experiment conducted in October 2012, as previously described (Gioia et al. 2015) and were chosen as representative of the majority of the diversity within the panel. Briefly, wheat genotypes were grown for 4 wk under high-N and nitrogen starvation (low-N) conditions in the Phytec Experimental Greenhouse at the Institute of Biosciences and Geosciences (IBG-2), Plant Sciences Institute, Forschungszentrum Jülich GmbH, Germany (50°54′36′′ N, 06°24′49′′ E). Seeds of uniform size and mass were visually selected, surface sterilized with 1% (*w/v*) NaClO for 15 min and pre-germinated. After germination, seedlings showing uniform growth (seminal root length, 1 to 2 cm) were transferred to soil-filled rhizoboxes, which were placed in the automated GROWSCREEN-Rhizo phenotyping system available at IBG-2. We used a Type 0 manually sieved peat soil (Nullerde Einheitserde; Balster Einheitserdewerk, Frondenberg, Germany), which provided low nutrient availability (ammonium N and nitrate N concentrations of <1.0 and <1.0 mg l^−1^, respectively). All plants were watered twice daily with 400 ml tap water and were supplied three times per week with 200 ml modified Hoagland solution (Hoagland and Arnon 1950), adapted for optimal N and N starvation conditions. Stock solution contained 5 mm KNO_3_, 5 mm Ca(NO_3_)_2_, 2 mm MgSO_4_, 1 mm KH_2_PO_4_, and trace elements. For N starvation conditions, KNO_3_ and Ca(NO_3_)_2_ were replaced with K_2_SO_4_ and CaCl_2_·6(H_2_O), respectively. The experiment was carried out under natural lighting in the greenhouse, with an air temperature of 18 to 24 °C and a relative humidity of 40% to 60%. For each N treatment, we used two replicates of each genotype with two plants per replicate (four plants per genotype in total). After 4 wk, leaves were pooled from two plants of the same genotype growing in the same rhizobox. Accordingly, four independent biological replicates (two replicates per N condition) were produced for each genotype, with the exception of wild emmer IG 46504, PI 233288, PI 466991, PI 538656, emmer MG 5293/1, and durum wheat Creso, Pedroso, and Trinakria, for which only three replicates were available, and emmer Molise Sel. Colli and durum wheat Simeto, for which eight replicates were available. The tissues were immediately frozen in liquid N_2_ and stored at −80 °C. Further details of the experiment and growth conditions are provided elsewhere (Gioia et al. 2015).

### RNA extraction and sequencing

RNA was extracted from 100 mg of frozen ground leaves per replicate using the Spectrum Plant Total RNA kit (Sigma-Aldrich, St Louis, MO, USA) followed by treatment with RNase-free DNase using the On-Column DNase I Digestion Set (Sigma-Aldrich). RNA integrity and purity were assessed by agarose gel electrophoresis and a Bioanalyzer 2100, respectively (Agilent/Bonsai Technologies, Santa Clara, CA, USA). Only RNA samples with an RNA integrity number >8.0 were considered suitable for analysis.

Library construction and RNA sequencing were carried out using the Illumina mRNA-Seq platform at the Montpellier Genomix sequencing facility (http://www.mgx.cnrs.fr) as previously described (David et al. 2014). Briefly, RNA samples were processed using TruSeq RNA sample preparation kits v2 (Illumina, San Diego, CA, USA). Libraries were quantified by RT-qPCR using the KAPA Library Quantification Kit for Illumina Sequencing Platforms (Roche, Basel, Switzerland), followed by quality control using a DNA 100 Chip on a Bioanalyzer 2100. Cluster generation and sequencing were carried out using the Illumina HiSeq 2000 instrument and TruSeq PE Cluster Kit v3, following the Illumina PE_Amp_Lin_Block_V8.0 recipe, and Illumina TruSeq PE Cluster v3-cBot-HS kits with the 2 × 100 cycles, paired-end, indexed protocol, respectively (David et al. 2014).

### RNA-Seq library processing and mapping

We pre-processed 128 raw paired-end RNA-Seq libraries (David et al. 2014). Cutadapt (Martin 2011) was then used to remove adaptor sequences and trim the end of reads with low-quality scores (parameter -q 20) while keeping reads with a minimum length of 35 bp. Reads with a mean quality score < 30 were discarded, and orphan reads (whose mates were discarded in the previous filtering steps) were also removed (David et al. 2014). The final quality of trimmed and filtered reads was assessed using FastQC (http://www.bioinformatics.babraham.ac.uk/projects/fastqc/).

The bread wheat (*Triticum aestivum* cv. Chinese Spring) genome assembly IWGSC RefSeq v2.1, along with the corresponding genome annotation, were downloaded from the IWGSC data repository hosted by URGI-INRAE (https://wheat-urgi.versailles.inra.fr/) and used as a reference to map each cleaned library to the A and B subgenomes. To validate our choice of reference genome, reads were also mapped to the available tetraploid wheat reference genomes: wild emmer accession Zavitan (https://www.ncbi.nlm.nih.gov/datasets/genome/GCA_002162155.3/) and durum wheat cultivar Svevo (https://www.interomics.eu/durum-wheat-genome). Pairwise genetic distances between the three reference genomes were computed using Mash v2.3 (Ondov et al. 2016).

STAR v2.7.0e (Dobin et al. 2013) was used for read mapping with the –quantMode TranscriptomeSAM and –quantTranscriptomeBan Singleend options. The output alignments were translated into transcript coordinates (in addition to alignments in genomic coordinates), allowing insertions, deletions, and soft-clips in the transcriptomic alignments. The transcriptomic alignments were used as inputs for salmon v1.6.0 (Patro et al. 2017) to quantify gene expression. Raw read counts were computed for all genes in each sample and, to filter out weakly expressed transcripts, only genes with at least 1 count per million (CPM) in at least 10 samples (of the same subspecies) were retained. This was calculated separately in each of the three subspecies and the raw counts of the filtered genes in each subspecies were then combined for downstream analysis (Supplementary Data Set 3).

### Variant identification

Variants were called by applying BCFtools v1.15 (previously SAMtools) (Danecek et al. 2021) to the alignment bam files. The “*bcftools mpileup*” command was used to determine the genotype likelihoods at each genomic position, with a minimum alignment quality of 20 and a minimum base quality of 30. The actual calls were obtained using the “*bcftools call*” command. The resulting VCF file was filtered using the “*bcftools view*” command, removing indels and keeping only sites covered by at least three reads in all genotypes. Subsequently, only biallelic SNPs with maximum values of 50% missingness and a 1% minor allele frequency were retained. To identify private and shared SNPs among the different subspecies, every possible comparison of the three subsampled VCF files (wild emmer, emmer, and durum wheat) was carried out using the “*bcftools isec*” command.

### Population genetics analysis

Variants were filtered (one SNP per 500 kb) using the VCFtools v0.1.17 *–thin 500000* option (Danecek et al. 2011) and then converted to ped format with PLINK v1.90p (Purcell et al. 2007). PLINK was also used to compute genetic distances between individuals with the *–distance-matrix* flag. The output matrix was used as input for PCA with the *cmdscale* function of R v4.2.1 (R Core Team 2022).

Genetic diversity statistics, including nucleotide diversity (*π* and *θ*) (Watterson 1975; Tajima 1983) were computed on the alignment bam files for each subspecies, from the folded site frequency spectra using ANGSD (Korneliussen et al. 2014). First, the *doSaf* function was used to estimate per-site allele frequencies (Saf); then, *realSFS* was used to get the site frequency spectra. The loss of diversity statistic (Vigouroux et al. 2002) was used to test the impact of primary and secondary domestication on the molecular diversity of the three subspecies. For primary domestication, the statistic was computed as [1−(*x*_emmer_/*x*_wild_)], where *x*_emmer_ and *x*_wild_ are the diversities in emmer and wild emmer, respectively, measured using *π* and *θ*. If *x*_emmer_ was higher than *x*_wild_, then the parameter was calculated as [(*x*_wild_/*x*_emmer_)–1]. The loss of diversity due to secondary domestication in durum wheat versus emmer was calculated as [1−(*x*_durum_/*x*_emmer_)], where *x*_durum_ and *x*_emmer_ are the diversities in durum wheat and emmer, respectively. If *x*_durum_ was higher than *x*_emmer_, then the parameter was calculated as [(*x*_emmer_/*x*_durum_)–1].

We calculated *F*_ST_ for each pair of populations using ANGSD (Korneliussen et al. 2014). Saf and 2D site frequency spectra were calculated for nucleotide diversity, and then, the *fst index* function was used to obtain the global estimate. To get an *F*_ST_ value for each gene in our dataset, we used the *fst print* function, which prints the posterior expectation of genetic variance between populations (called A), and total expected variance (called B) for every locus. We then computed the weighted *F*_ST_ as the ratio between the summed As and summed Bs for every gene region, using an ad hoc R script.

### Expression profiles, heritability, and *Q*_ST_ analysis

Raw read counts of the 32,358 genes were normalized using the *vst* method allowing the additive CV_A_ (standard deviation/mean) to be calculated for the two N conditions in every subspecies, averaging the biological replicates of every genotype. The statistical loss approach (Vigouroux et al. 2002) was then applied to test the loss of expression diversity in the different groups, as previously reported (Bellucci et al. 2014). The statistical significance of the differences between each CV_A_ value and the percentage loss of expression diversity was determined using the Mann–Whitney *U*-test in R v4.2.1 (R Core Team 2022) with the function *wilcox.test*.

To compute heritability, the raw counts of each subspecies under each condition were first normalized using the trimmed mean *M*-values normalization method in the R package edgeR (Robinson et al. 2010) and the voom normalization method in the R package limma (Smyth 2005). To determine the variance component of each factor and heritability, the following model was considered:


$$Y_{\mathrm{ijkl}}=S_{i}+G_{j(i)}+N_{k}+(S\times N{)}_{\mathrm{ik}}+(G\times N{)}_{\mathrm{jk}(i)}+\varepsilon_{l(\mathrm{ijk})}$$

where $Y_{\mathrm{ijkl}}$ is the normalized gene expression level, $S_{i}$ is the species factor, $G_{j(i)}$ is the genotype factor nested in species, $N_{k}$ is the N-level factor, $(S\times N{)}_{\mathrm{ik}}$ is the interaction between species and N levels, $\;(G\times N{)}_{\mathrm{jk}(i)}$ is the interaction between genotypes and N levels, and $\varepsilon_{l(\mathrm{ijk})}$ is the residual error. All factors were treated as random effects in the model except the intercept, which was a fixed effect. The linear mixed models were fitted using the *lmer* function in R package lme4 based on the normalized data of each transcript (Bates et al. 2015). The heritability (*H*^2^) was calculated as $H^{2}=\frac{V_{S}+V_{G}}{V_{A}}$, where $V_{A}=V_{S}+V_{G}+V_{N}+\frac{V_{S\times N}}{n}+\frac{V_{G\times N}}{n}+\frac{V_{\varepsilon }}{n}$, $V_{S}$ is the variance of species, $V_{G}$ is the variance of genotype, $V_{N}$ is the variance of N level, $V_{S\times N}$ is the variance of species and N-level interaction, $V_{G\times N}$ is the variance of genotype and N-level interaction, $V_{\varepsilon }$ is the residual variance, and *n* is the number of N levels. $V_{S\times N}$ and $V_{G\times N}$ represent the genotype × environment interaction variance components at the species and genotype (nested in species) levels, respectively.

*Q*_ST_ was calculated between pairs of the three subspecies under low-N and high-N conditions separately. The wild emmer *vs* emmer comparison revealed the effects of primary domestication, the emmer *vs* durum wheat comparison revealed the effects of secondary domestication, and the wild emmer *vs* durum wheat comparison revealed the cumulative effect of domestication. To this end, the model can be reduced to $\;Y_{\mathrm{ijl}}=S_{i}+G_{j(i)}+\varepsilon_{l(\mathrm{ij})}$ at each N level. The *Q*_ST_ value was calculated as $Q_{\mathrm{ST}}=\frac{V_{S}}{V_{S}+V_{G}}$, that is, the ratio of between-species to within-species variances.

*Q*_ST_ Distributions were used to perform a “selection scan” on a restricted number of genes. First, genes were filtered for *H*^2^ ≥ 0.7, and in order not to lose genes whose expression was strongly influenced by N availability, the species × environment (*S × N*) variance component was also evaluated (i.e. every species subgroup × N condition), retaining those genes meeting the threshold *S × N* ≥ 0.2 (Supplementary Fig. S3). Successively, we obtained six different *Q*_ST_ value distributions (*Q*_ST WILD EMMER *VS* EMMER_, *Q*_ST EMMER *VS* DURUM WHEAT_, and *Q*_ST WILD EMMER *VS* DURUM WHEAT_, each for high-N and low-N conditions), and we retained the 5% right-hand tail of every distribution. Finally, we compared *F*_ST_ and *Q*_ST_ values for every gene, discarding *F*_ST_ values < 0.01. We confirmed that every retained gene satisfied the condition *Q*_ST_ > *F*_ST_, allowing it to be classed as undergoing directional selection. For these selected genes, SNPs were annotated using SnpEff v.5.1d (Cingolani et al. 2012).

### Differential expression analysis

Differential gene expression was assessed by analyzing the pre-processed raw count dataset (32,358 genes). We identified DEGs by comparing (i) two conditions (i.e. high-N and low-N levels) within each subspecies, and (ii) pairs of the three subspecies under the same N levels, which considered the genotypes nested in species. For the two scenarios, we used three different approaches to detect DEGs: one linear model-based approach implemented in the R package limma (Smyth 2005), and two Poisson model-based approaches implemented in the R packages edgeR (Robinson et al. 2010) and DESeq2 (Love et al. 2014). In all approaches, the normalization of raw counts was applied by default in the package before differential analysis. To reduce the number of false positives, the intersection of DEGs resulting from the three approaches was retained (Rapaport et al. 2013; Zhang et al. 2014), and the significance threshold was set to an adjusted *P*-value < 0.001. The DEGs between high- and low-N levels in at least one subspecies were used for PCA following the *DESeq2* approach (Love et al. 2014), first using all the DEGs, then repeating the analysis on the DEGs considered to be under selection. At each step, counts were normalized using the *vst* method before the *plotPCA* function was applied to define principal components 1 and 2 for the two N levels separately. The expression patterns of the DEGs considered to be under selection were plotted from normalized read counts using the R package pheatmap (https://github.com/raivokolde/pheatmap).

### GO enrichment analysis

Enriched terms in the DEGs and genes under selection were identified using agriGO v.2.0 (Tian et al. 2017). All annotated genes of bread wheat were used as background, and the following parameters were set: hypergeometric test, multiple hypothesis test adjustment according to the Hochberg FDR procedure at significance level < 0.05, and minimum number of mapping entries = 3.

### Accession numbers

The RNA-Seq libraries generated and analyzed in this study have been deposited in the Sequence Read Archive (SRA) of the National Center of Biotechnology Information (NCBI) under BioProject number PRJNA1015013. The nucleotide sequences of all discussed genes are available on the GarinGenes Database (https://wheat.pw.usda.gov/cgi-bin/GG3/browse.cgi) and can be accessed using the Gene model IDs provided in the Supplementary Data Sets (e.g. *TraesCS2A03G0941300*).

## Supplementary Material

koae202_Supplementary_Data

## Data Availability

Scripts used in this study are available at Github https://github.com/PapaLab/transcriptomics_triticum.git.

## Supplementary data

The following materials are available in the online version of this article:

**Supplementary Figure S1.** Folded site frequency spectra (SFS) of single-nucleotide polymorphisms (SNPs) in the three wheat *taxa*.

**Supplementary Figure S2.** Principal component analysis (PCA) of 32 wheat genotypes based on single-nucleotide polymorphisms (SNPs) using different reference genomes.

**Supplementary Figure S3.** Workflow of gene expression selection scanning.

**Supplementary Figure S4.** Comparison of Q_*ST*_ and F_*ST*_ estimates of the 5,868 genes showing *H*^2^ ≥ 0.7 or *S × N* ≥ 0.2.

**Supplementary Figure S5.** Gene ontology (GO) categories of genes under selection.

**Supplementary Figure S6.** Genome-wide distribution of differentially expressed genes (DEGs) in the comparison between contrasting N conditions within each subspecies.

**Supplementary Figure S7.** GO Classification of DEGs in the comparison between contrasting N conditions within each subspecies.

**Supplementary Figure S8.** DEGs between subspecies.

**Supplementary Figure S9.** Expression profiles of the 101 DEGs putatively under selection in the three wheat *taxa* in high-N (N+) and low-N (N−) conditions.

**Supplementary Figure S10.** Principal component analysis (PCA) of 32 wheat genotypes based on expression data of all 32,358 genes in each subspecies.

**Supplementary Table S1.** Mean CV_A_ in gene expression for the three wheat *taxa* and loss of expression diversity for two gene subgroups (6,991 differentially expressed and 25,367 non-differentially expressed genes).

**Supplementary Table S2.** Synonymous and non-synonymous mutations and non-synonymous/synonymous ratios of two gene groups: 967 genes under selection and 967 randomly selected genes.

**Supplementary Data Set 1, A to C.** List of the 128 samples and reads mapping results using three different reference genomes.

**Supplementary Data Set 2.** Results of single-nucleotide polymorphism (SNP) calling and nucleotide diversity estimates, using three different reference genomes.

**Supplementary Data Set 3.** Raw read counts of the 32,358 genes in the 128 RNA-Seq samples.

**Supplementary Data Set 4.** List of the 967 genes retained from the “selection scan”.

**Supplementary Data Set 5, A to D.** List of GO “Biological process” and “Molecular function” subcategories for upregulated and downregulated DEGs under different nitrogen N conditions for each subspecies.

**Supplementary Data Set 6.** Functional annotations of the DEGs between N conditions in each subspecies. Genes with the top 5% |log_2_FC| values are shown.

**Supplementary Data Set 7.** Functional annotations of the DEGs between subspecies under all N conditions.

**Supplementary Data Set 8.** Functional annotation of the 101 genes selected by the integration of selection signatures and differential expression analysis between N conditions.
