# Supplementary material for: Transcriptomic response to nitrogen availability reveals signatures of adaptive plasticity during tetraploid wheat domestication
Source: Plant Cell. 2024 Jul 26;36(9):3809–23. doi: 10.1093/plcell/koae202 (PMC11371143; doi:10.1093/plcell/koae202)
Supplement: koae202_Supplementary_Data [file koae202_supplementary_data.zip › Supplementary_Data.pdf]

Supplementary Figures

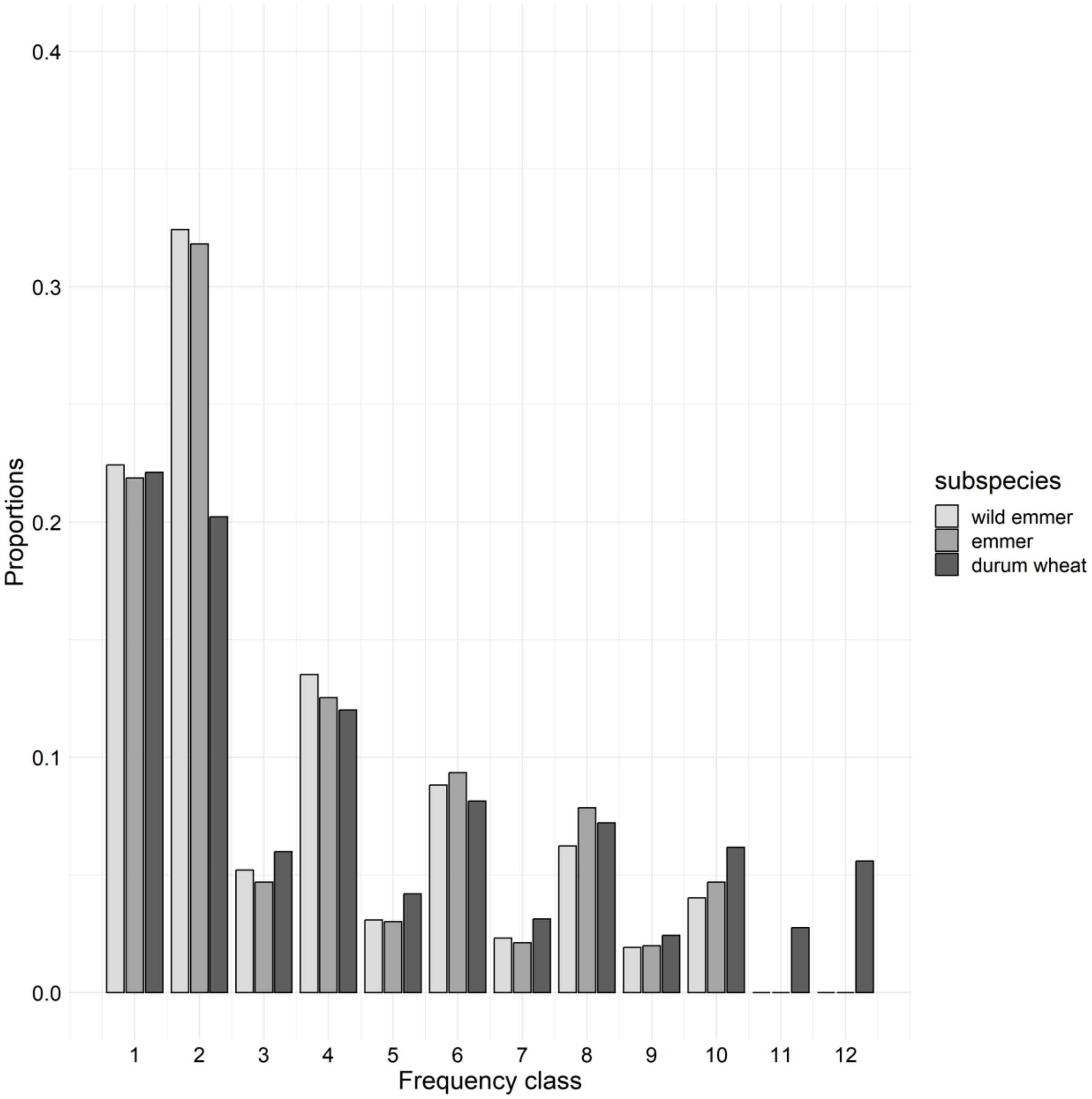

Supplementary Figure S1: Folded site frequency spectra (SFS) of single nucleotide polymorphisms (SNPs) in the three wheat *taxa*. Each column indicates the abundance of SNPs that fall into a particular frequency class and the three colors represent different *taxa*. (Supports Supplementary Data Set S2).

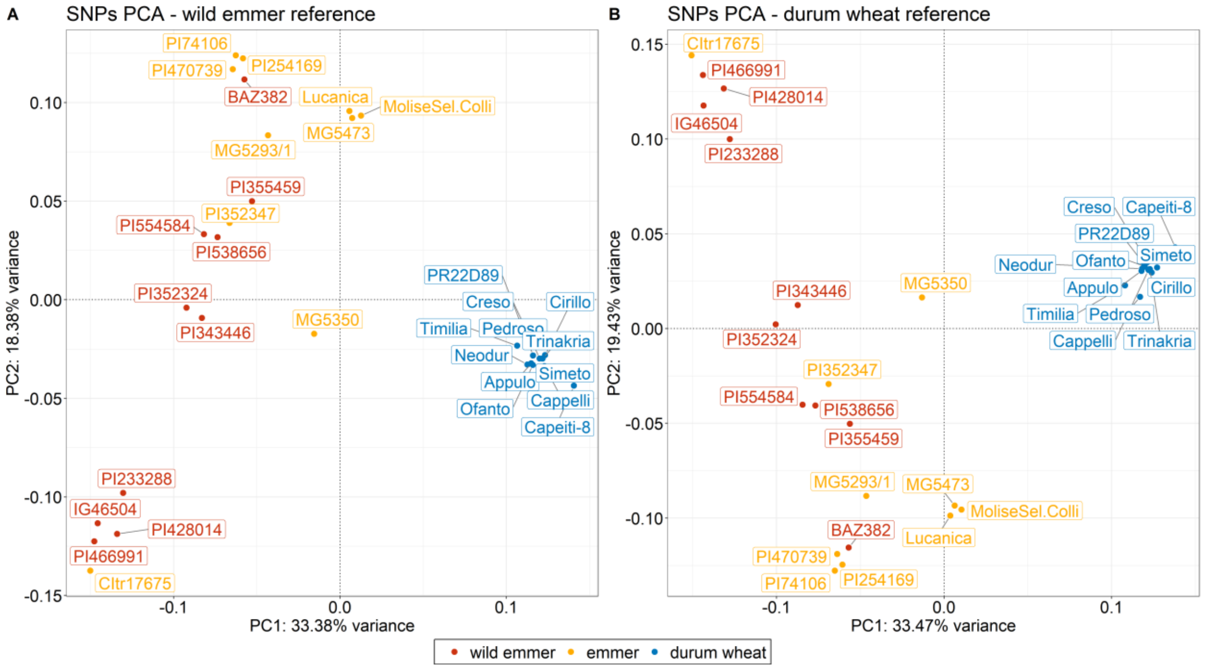

Supplementary Figure S2: Principal component analysis (PCA) of 32 wheat genotypes based on single-nucleotide polymorphisms (SNPs) using different reference genomes. A Using wild emmer reference. **B** Using durum wheat reference. The first two principal components (PC1 and PC2) are shown. The three colors represent different *taxa*. Labels show the accession name of each genotype. (Supports Figure 1).

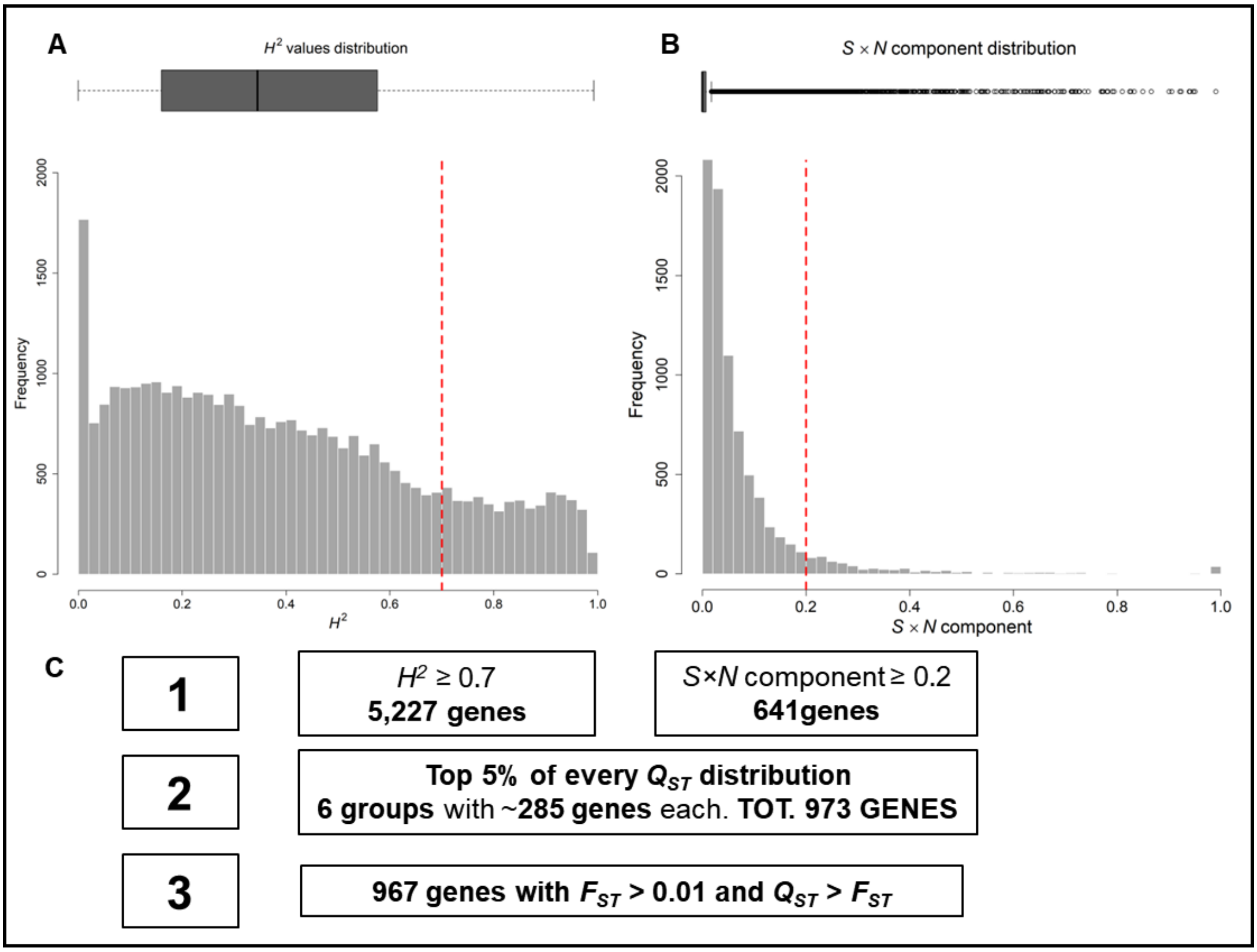

Supplementary Figure S3: Workflow of gene expression selection scanning. Histograms and boxplots showing distributions of **A** H^2 values and **B** *S*×*N* components calculated using all 32,358 genes. Red dashed lines indicate the arbitrary thresholds chosen for each distribution as the first filtering step of the selection scan. **C** Subsequent steps for the identification of gene expression selection signatures. (Supports Supplementary Data Set S4).

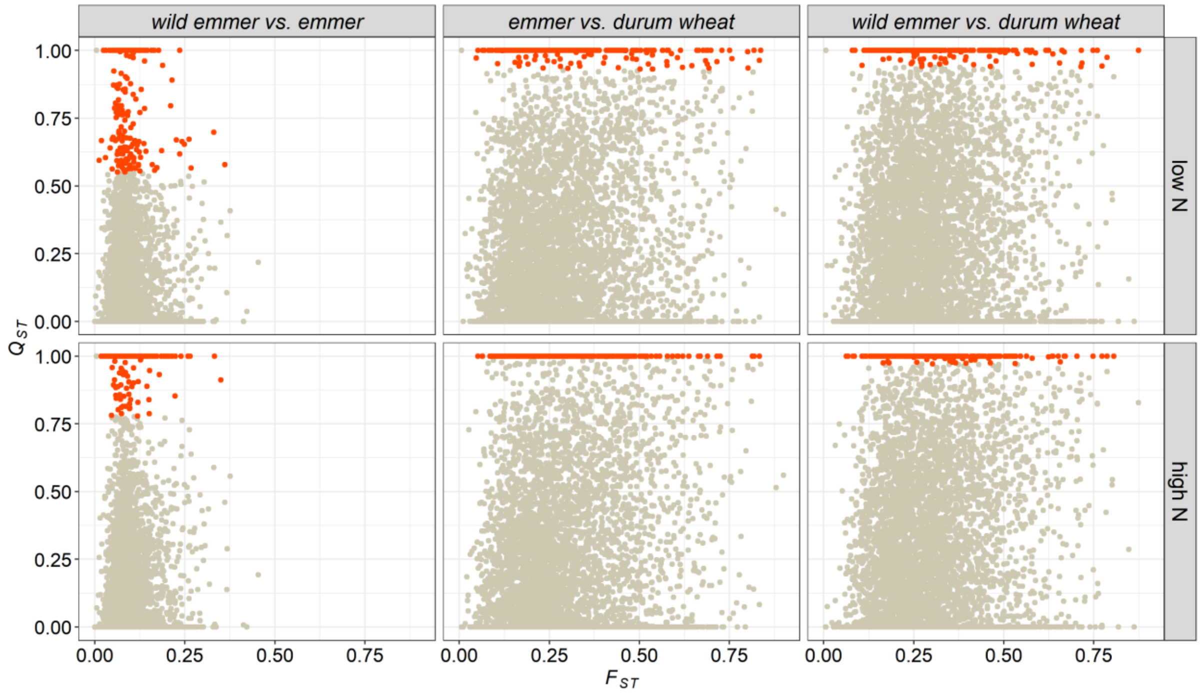

Supplementary Figure S4: Comparison of Q_{ST} and F_{ST} estimates of the 5,868 genes showing H^2 ≥ 0.7 or *S×N* ≥ *0.2*. Q_{ST} *vs.* F_{ST} values are shown for every subspecies pairwise comparison under low-N and high-N conditions. Orange dots represent the genes in the genes in the 5% right-hand tail of the Q_{ST} distributions and were considered as candidates for selection. (Supports Figure 3).

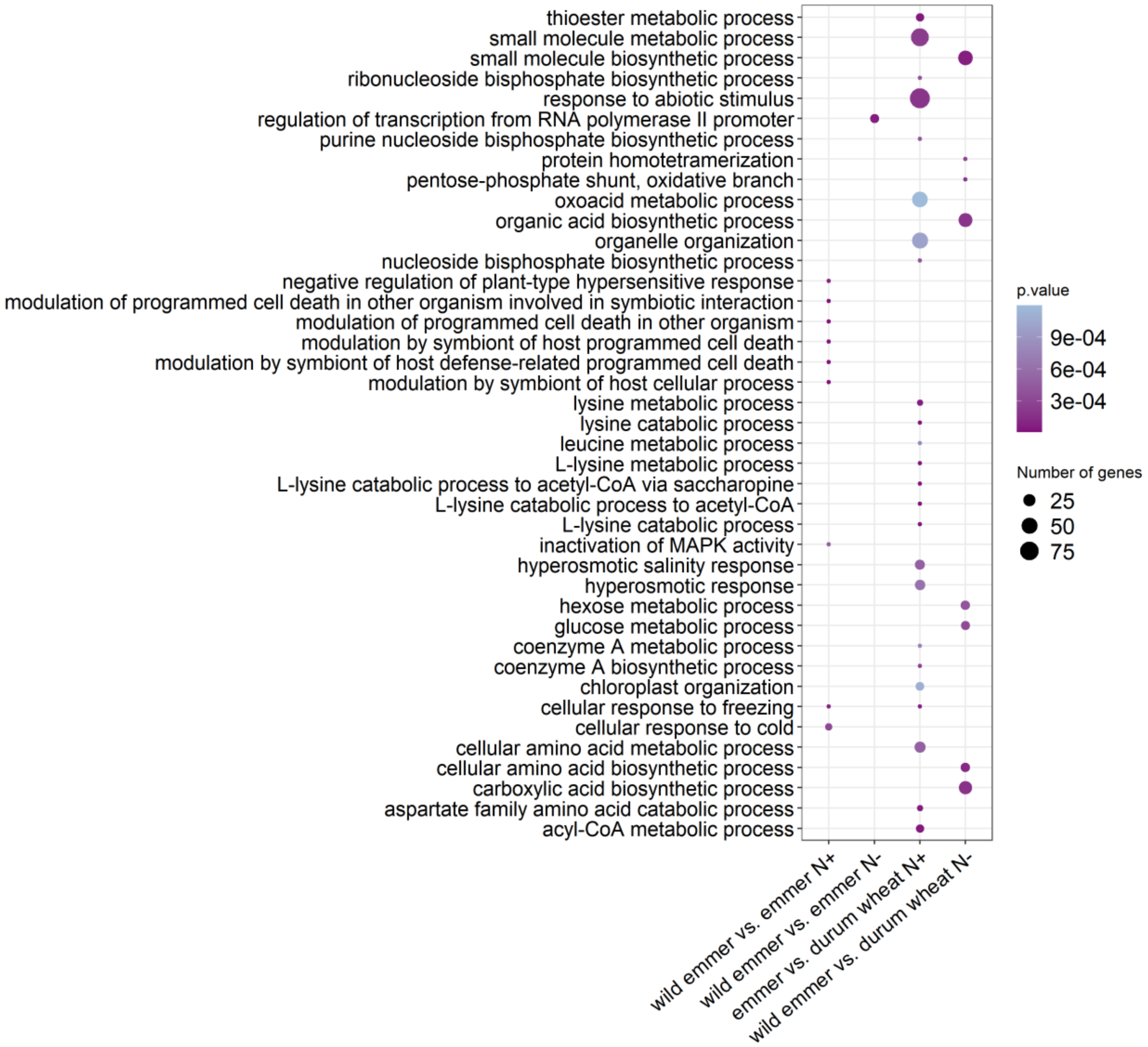

Supplementary Figure S5: Gene Ontology (GO) categories of genes under selection. Dot plot showing the GO categories enriched in each group of genes putatively under selection during primary domestication (wild emmer to emmer), secondary domestication (emmer to durum wheat) and both processes (wild emmer to durum wheat) in high-N (N+) and low-N (N–) conditions. The size of the dots represents the number of genes associated with the GO term and colors indicate the p-values. The represented categories passed the multi-test adjustment Hochberg FDR < 0.05. (Supports Supplementary Data Set S4).

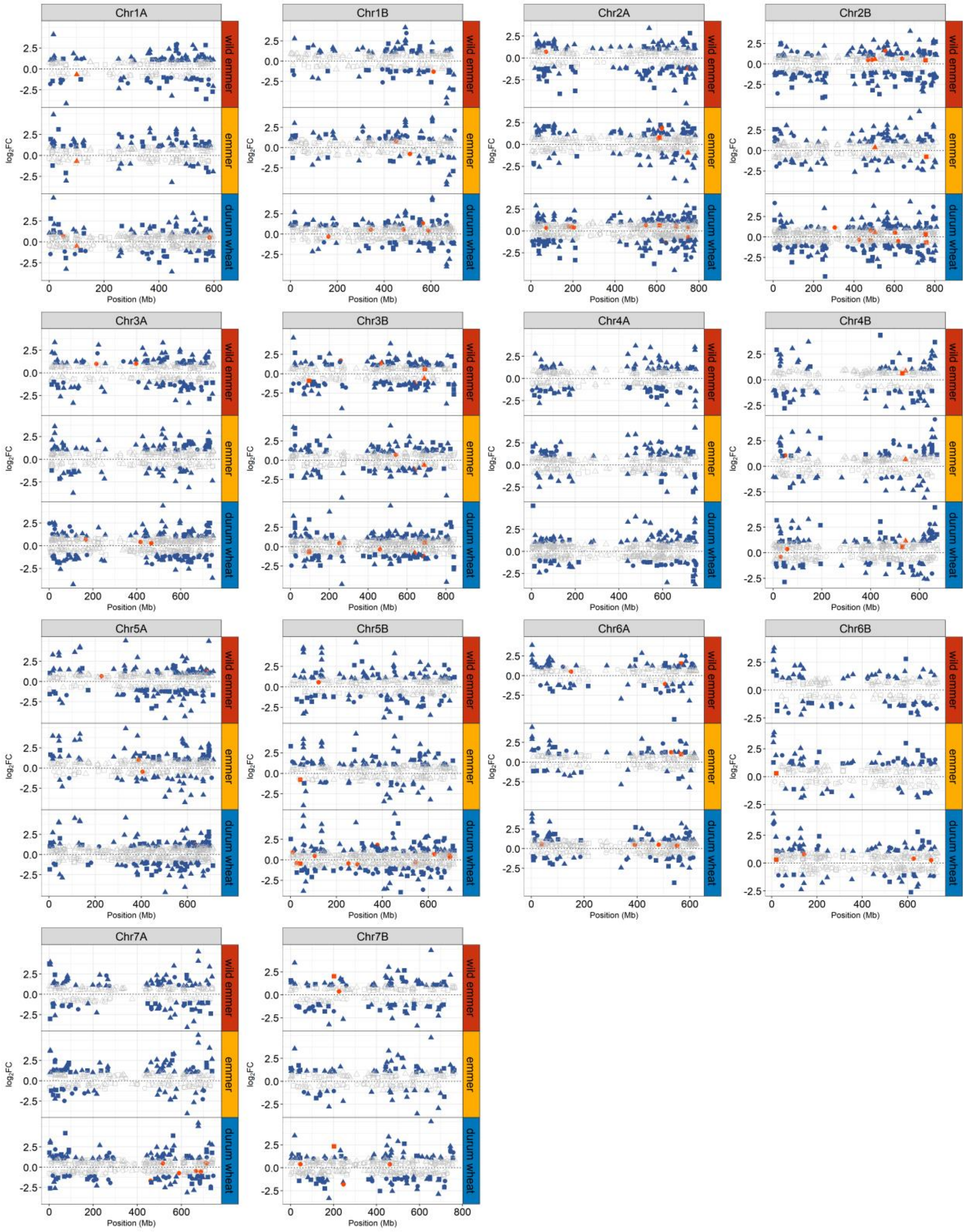

Supplementary Figure S6: Genome-wide distribution of differentially expressed genes (DEGs) in the comparison between contrasting N conditions within each subspecies. Circles represent private DEGs, squares represent DEGs shared between two subspecies, and triangles represent DEGs common to all three subspecies. Full blue shapes represent DEGs satisfying the threshold | log_2FC | ≥ 1. Gray empty shapes represent DEGs satisfying the threshold | log_2FC | ≤ 1. DEGs putatively under selection are shown in orange. (Supports Figure 4).

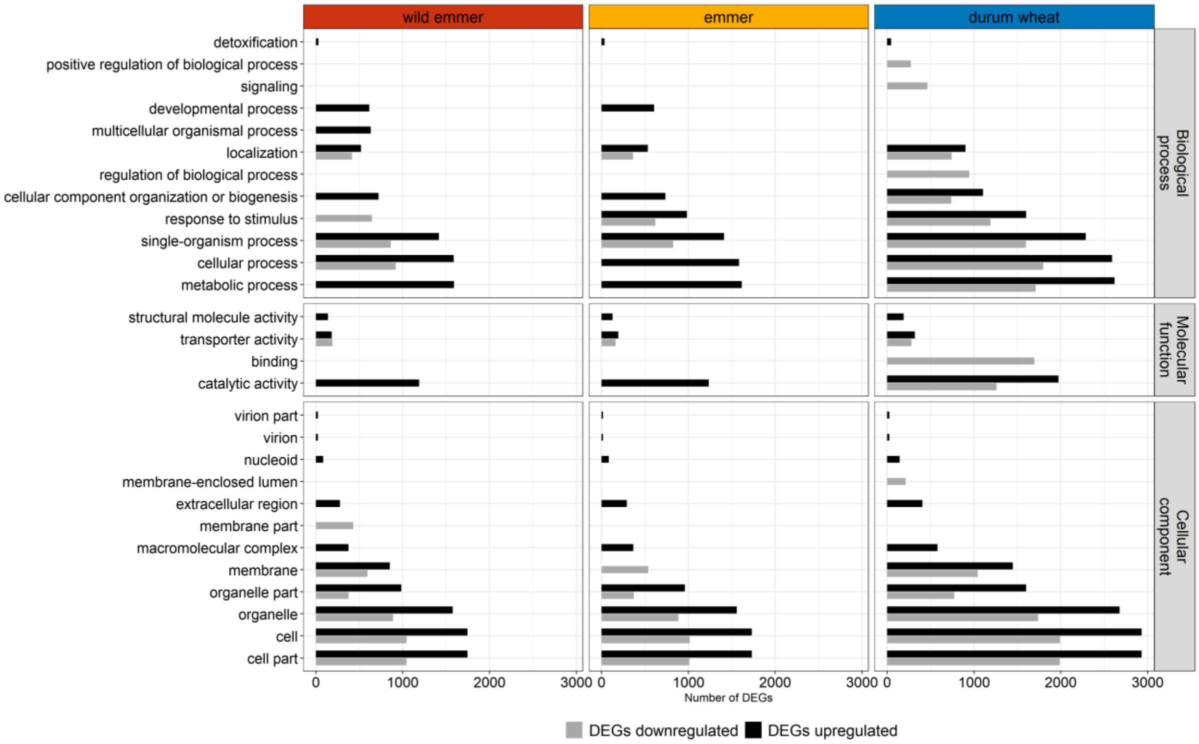

Supplementary Figure S7: GO classification of DEGs in the comparison between contrasting N conditions within each subspecies. Black and gray bars indicate the number of upregulated and downregulated DEGs, respectively. The length of the bars represents the number of DEGs associated with each GO term. The represented categories passed the multi-test adjustment Hochberg FDR < 0.05. (Supports Supplementary Data Set S5).

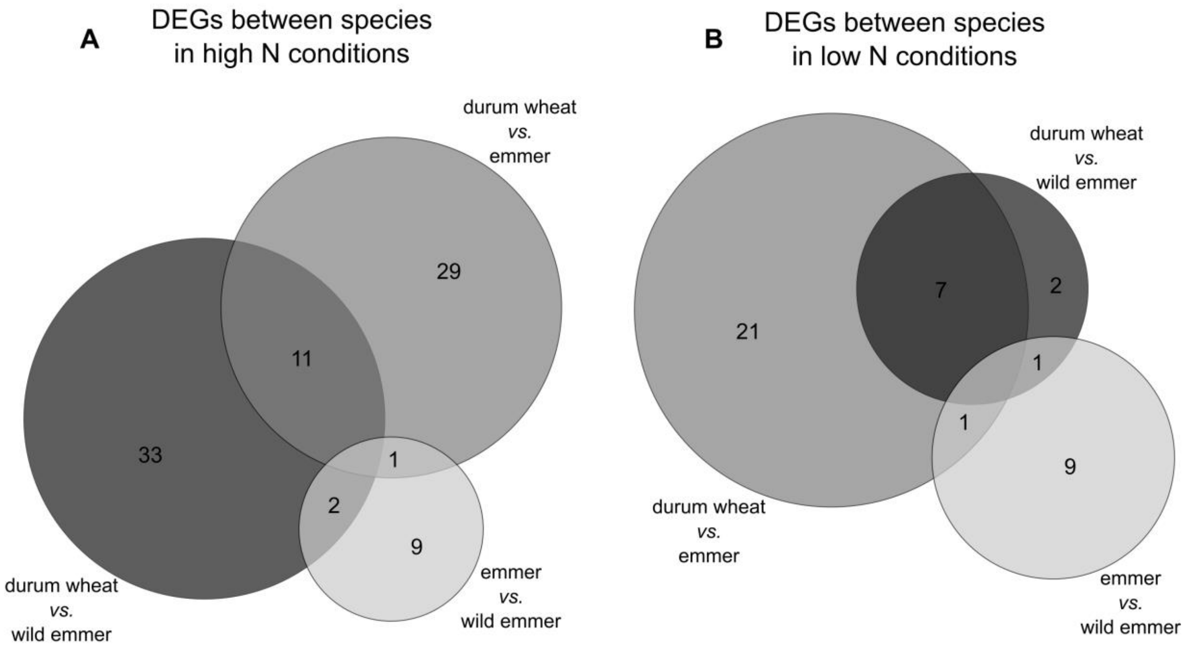

Supplementary Figure S8: DEGs between subspecies. Venn diagrams show the numbers of DEGs between each subspecies in pairwise comparisons under **A** high-N conditions and **B** low-N conditions. (Supports Supplementary Data Set S7).

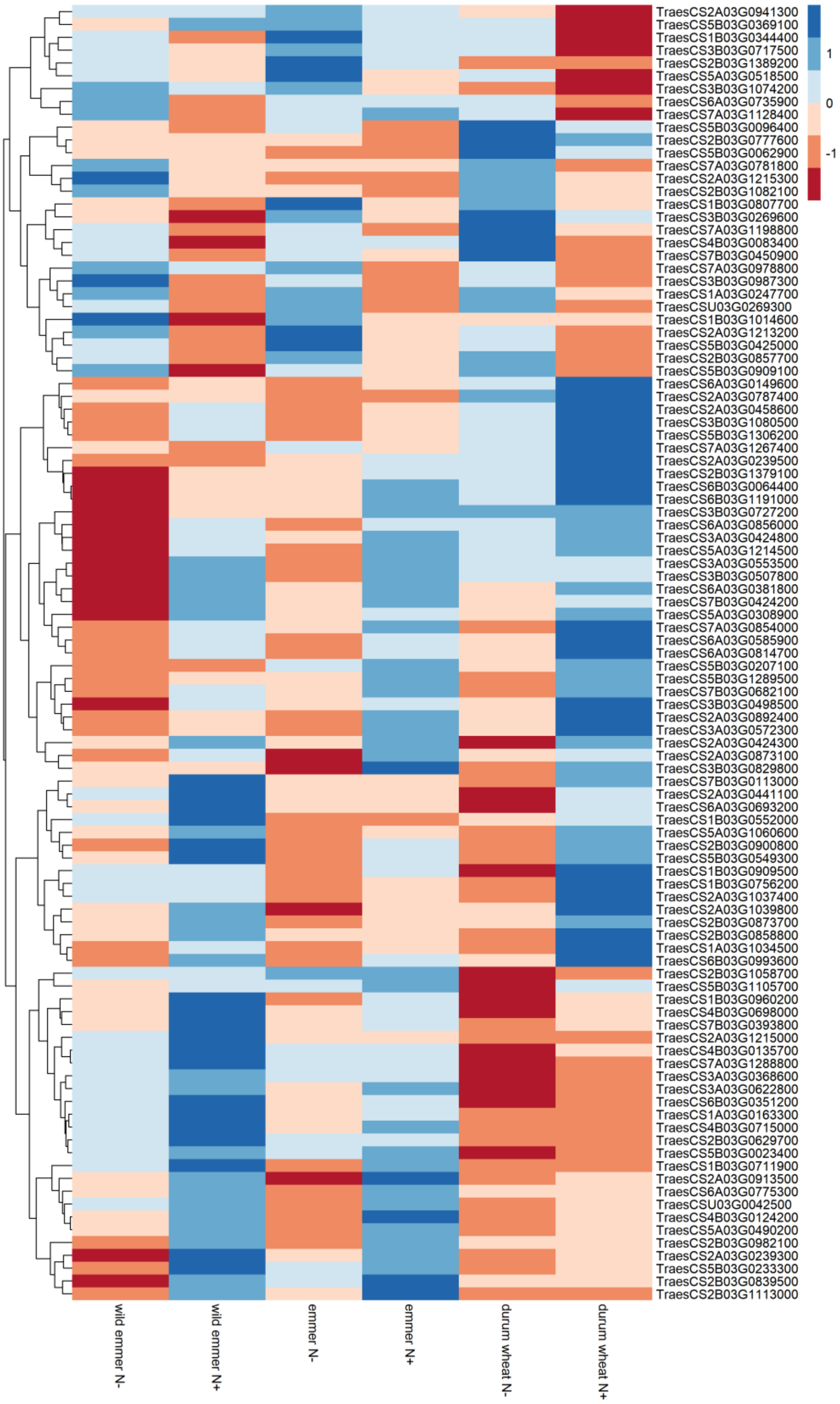

Supplementary Figure S9: Expression profiles of the 101 DEGs putatively under selection in the three wheat *taxa* in high-N (N+) and low-N (N–) conditions. Normalized read counts were rescaled in Z-Score to better compare trends across samples. Red colors indicate low expression levels, blue colors high expression. Each row represents data for one gene. (Supports Figure 5 and Supplementary Data Set S8).

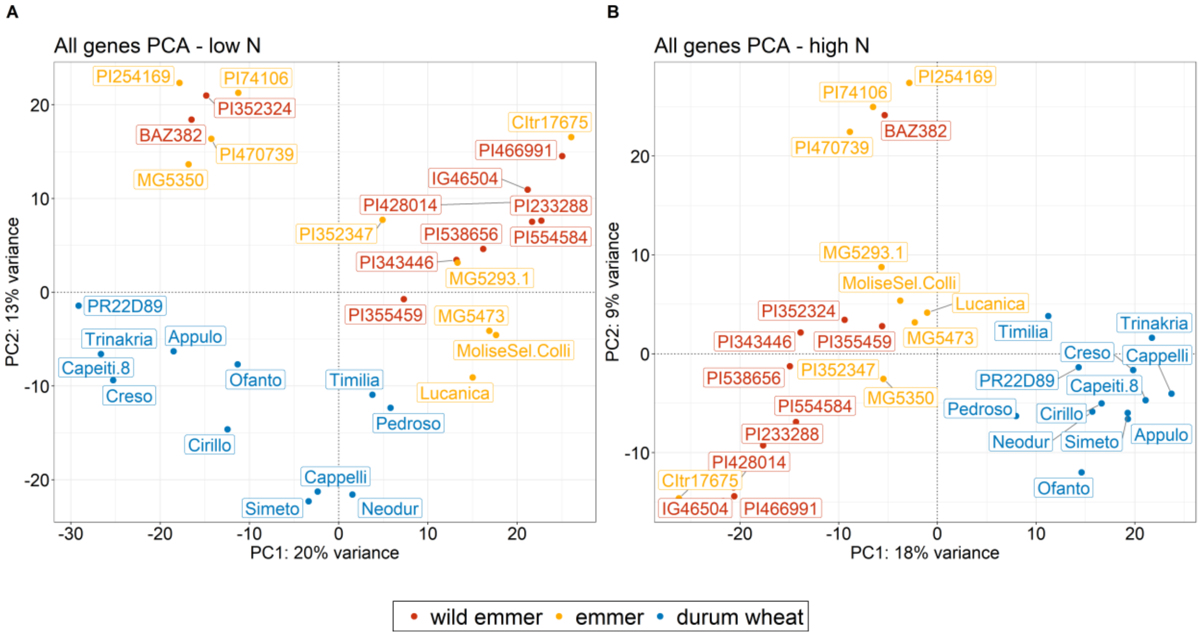

Supplementary Figure S10: Principal component analysis (PCA) of 32 wheat genotypes based on expression data of all 32,358 genes in each subspecies. A low-N conditions and **B** high-N conditions. Samples are represented by *taxon*-specific colored dots. Labels show the accession name of each genotype. (Supports Figure 5).

Supplementary Tables

					Loss of expression diversity (%)		
		**Wild emmer**	**Emmer**	**Durum wheat**	Lpd	Lsd	Both
6,991 DEGs	CV_A high N	0.057	0.052	0.042	8.8	18.3*	25.4
	CV_A low N	0.072	0.061	0.052	15.1*	14.5*	27.4
25,367 NON-DEGs	CV_A high N	0.062	0.056	0.048	9.7	14.3*	22.6
	CV_A low N	0.074	0.061	0.054	17.6*	11.5*	27.0

Supplementary Table S1: Mean CV_A in gene expression for the three wheat taxa and loss of expression diversity for two gene subgroups (6,991 differentially expressed and 25,367 non-differentially expressed genes). Diversity loss is shown during primary domestication (wild emmer to emmer, Lpd), secondary domestication (emmer to durum wheat, Lsd) and both processes (wild emmer to durum wheat).
*$p < 0.001$, Mann–Whitney U-test for difference between Lpd and Lsd within each N condition, and difference between high N and low N within Lpd and within Lsd.

	**967 genes under selection**			**967 genes randomly selected**		
	**Wild emmer**	**Emmer**	**Durum wheat**	**Wild emmer**	**Emmer**	**Durum wheat**
Mean Synonymous mutations	3.67	3.47	2.09	4.77	4.67	3.41
Mean Non-synonymous mutations	2.87	2.71	1.44	3.83	3.85	2.57
Mean Non-synonymous / synonymous	0.78	0.78	0.69	0.80	0.82	0.76*

Supplementary Table S2: Synonymous and non-synonymous mutations and non-synonymous/synonymous ratios of two gene groups: 967 genes under selection and 967 randomly selected genes. Mean values are shown for the three wheat taxa.
*$p < 2.2e-16$, Kolmogorov-Smirnov, two-sided test for difference in non-synonymous/synonymous ratios between genes under selection and randomly selected genes within each *taxon*.
