# Supplementary material for: Transcriptomic response to nitrogen availability reveals signatures of adaptive plasticity during tetraploid wheat domestication
Source: Plant Cell. 2024 Jul 26;36(9):3809–23. doi: 10.1093/plcell/koae202 (PMC11371143; doi:10.1093/plcell/koae202)
Supplement: koae202_Supplementary_Data [file koae202_supplementary_data.zip › tpc.00929.2023-s07.pdf]

THE PLANT CELL

Transcriptomic Response to Nitrogen Availability Highlights Signatures of Adaptive Plasticity During Tetraploid Wheat Domestication

Alice Pieri, Romina Beleggia, Tania Gioia, Hao Tong, Valerio Di Vittori, Giulia Frascarelli, Elena Bitocchi, Laura Nanni, Elisa Bellucci, Fabio Fiorani, Nicola Pecchioni, Stefania Marzario, Concetta De Quattro, Antonina Rita Limongi, Pasquale De Vita, Marzia Rossato, Ulrich Schurr, Jacques David, Zoran Nikoloski, and Roberto Papa

Review Timeline:

Submission Date:	10-Oct-2023
Editorial Decision:	21-Nov-2023
Revision Received:	25-Apr-2024
Editorial Decision:	04-Jun-2024
Revision Received:	18-Jun-2024
Accepted:	28-Jun-2024

Prof. Roberto Papa
Department of Agricultural, Food and Environmental Sciences, Marche Polytechnic University
Department of Agricultural, Food and Environmental Sciences
Ancona 60131
Italy

Dear Roberto / Dr. Papa:

We have received reviews of your manuscript entitled "Transcriptomic Response to Nitrogen Availability Highlights Signatures of Adaptive Plasticity During Tetraploid Wheat Domestication." Thank you for submitting your best work to The Plant Cell. The editorial board agrees that the work you describe is substantive, falls within the scope of the journal, and may become acceptable for publication, pending revision and potential re-review.

However, we should stress that we are reluctant to see manuscripts undergoing multiple rounds of revision and would be unlikely to offer you more than one chance to satisfy the reviewers.

Please contact us if there are ambiguous comments or if you wish to discuss the revision.

In particular, the editors urge you to pay attention to ways that may address or mitigate the concerns the reviewers raise about bias in the underlying SNP dataset as well as the degree of explanation, testing, and validation associated with the Qst-Fst methodology.

Given the nature of the comments, we are offering you 60 days from when we have issued this decision to complete the revision. If a revision is not returned within this time frame, and if you have not been granted an extension, we will withdraw the manuscript, which will leave you free to submit the work elsewhere. If you need an extension, we encourage you to contact us at any point before the 60 days have passed.

When you are ready to submit the revised version, please upload a highlighted copy that indicates all changes made in response to the editor and reviewer recommendations. Include an itemized list of all changes made in response to each of the reviewer's suggestions in the "Response to Reviewers" section; please note that reviewers do not have access to your cover letter, nor was this decision letter shared with them.

Thank you for the privilege of reviewing your work. We look forward to receiving your revised manuscript.

Sincerely,

The Plant Cell Board of Editors

Please note the following:

-The Plant Cell now requires authors to complete and submit an author revisions checklist upon submission of a revised manuscript. The aim of the checklist is to aid authors in preparing a high-quality manuscript, facilitate the review and assessment of revised manuscripts, and help to ensure that journal standards are maintained across the board. If your manuscript is accepted, the completed checklist will be published as supplemental material attached to the article online. Please download a copy of the checklist (pdf fillable form) at this link, for submission with your revised manuscript: https://tpc.msubmit.net/html/Author_Revisions_Checklist.pdf.

-Supplemental materials should be restricted to large datasets and tables, presentation of replicates, and validation of reagents, methods, or genotypes. Any data that are used to support the major claims must be in the main manuscript. Supplemental figure legends must indicate what figure in the main manuscript is supported by the supplemental data presented. Please justify how each of the supplemental figures meet the criteria.

-Sampling methods and nature of "biological replicates" should be described precisely (i.e. different plants, parts of plants, pooled tissue, independent pools of tissue, sampled at different times, etc), along with a clear description of and rationale for any statistical analyses conducted. The reader should know exactly what was sampled; what forms the basis of the calculation of any means and statistical parameters reported. This is also necessary to ensure that proper statistical analysis was conducted.

-Want to add this revision deadline to your calendar? Click below!

-- Reviewer comments:

Reviewer #1 (Comments for the Author):

In this manuscript the authors use a large transcriptomic dataset coupled with experimental work across a range of wheat-realated species to answer very interesting quiestions about ecolution and adaptation. While the manuscript presents an interesting concept and may provide a unique contribution to the field, it seems to me that it suffers from some methodological issues that prevent its publication in its current form. I detail my criticisms below; the authors should take them in consideration and make sure to adjust their claims to what is actually supported by the data. The english language should be improved. Here below, my major concerns; below, line by line comments.

##Major issues:

There are two components in this manuscript: one relative to SNP diversity and one to gene expression.
While interesting and quite innovative, I believe that the former is flawed or at least impaired by underling bias with the data. This all traces back to the reference genome used to call SNPs. Being bread wheat (a choice that I do not agree with, see below), I believe that it is expected that the diversity observed in the dataset follows a gradient going from cultivated (low distance from bread wheat, low diversity) to wilds (high distance from bread wheat, high diversity). This is not to say that this is not generally true, as indeed it is reported by many studies, but rather that it is not supported by the data. As things stand, diversity is not assessed in an even manner; it is biased by looking through the lens of the bread wheat genome. This bias is not desirable nor necessary, since high quality genome sequences are available for the species under study. If the authors wish to report on the SNP diversity, then I suggest supporting the findings by a new alignment and SNP calling for each of the species; once that is done, it is possible to compute measures of nucleotide diversity among individuals of each species.
The second component of the manuscript, that is transcriptomics, is nicer and potentially very relevant, although it can also be improved quite substantially (see more specific comments below). Also in this case, I wonder how the results may be biased by the fact that the authors chose to use bread wheat as reference genome. The explanation given in L471 did not convince me and I would suggest to rather use durum wheat as a reference, if not multiple references as required by my point above.

##Other general and important comments:

This is an interesting experimental setup, but I wonder to what extent N availability can be put in relation to domestication (primary or secondary). Is there any information about use of fertilizers in association with early farming and how this aligns with the timeline of domestication? It seems a bit stretched to put in relation N availability to genomic signatures of selection across domestication, unless more context is provided to justify this hypotesis.

If I got it right, variants were called on transcriptomic data. Authors need to discuss the implications of this approach, especially since these variants come from alignment to a species not included in the dataset. Are these variants causative of the adaptation or rather they are in linkage with upstream or downstream variants which may play a role? This should be acknowledged. If SNP calling gets improved as per my suggestions above, it would be quite relevant to look for synonymous/non synonymous exonic variants in the dataset to further support the interpretation of evolutionary processes in relation to N use. This would enrich the GO analysis that, as it stands, is quite general and not very convincing.

##Line by line comments
Line by line comments
L31. Genotypes or species? Say a few words to specify this.
L33. Refrain from using jargon in abstract. A naïve reader may not know what Qst is. Same for primary and secondary domestication.
L36 Nitrogen what? Quantity? Availability?
L66-67 it seems that there is a logic jump over here. How phenotypic plasticity relates to the previous paragraph? Rephrase.
L69 this sentence reads a bit controversial; I believe that SDGs are aligned with a *reduction* of fertilizer use.
L93 provide data specific for each species (see comment about methods)
L97-100 although it is certainly true that DW diversity is lower than that of wilds, I think that this is not necessarily the only explanation as your SNP data will be significantly biased towards cultivated wheat. Simply, DW is more similar to BW that you are using as a reference and hence you detect less SNPs. I expect a similar pattern in mapping % (see comment above)

L103-106 same as above; this claim, although possibly true, results from a biased dataset. If ones would like to compare the amount of diversity available in each of the three species, assuming that the selection of genotypes is representative of the species, this should be done on SNPs called on the respective reference genomes.
L115 see comment above
L137 I am not sure what is the meaning of "nucleotide diversity in this context"
L141 this is a very interesting result, but I don't see how it relates to the association between domestication and diversity reported in L142-144
L146 interesting plots; perhaps a log scale would allow to see differences between species better?
L183 VERY strong claim, given the limitations discussed above and below. I suggest to tone down a bit here and elsewhere.
L190-191 I would argue that this is still no proof of "representativeness" of the collection. I suggest discussing this aspect in more detail, noting that a limitation of the study is the limited number of genotypes considered (although for good reasons that I fully understand).
L223-267 This are a nice stories but I wonder if the data really support an interpretation going this far. It seems quite speculative given the type of data and genetic materials; I would reduce this part and note it as speculation.
L305 reference formatting is odd here
L309 provide more details about which genes and how many, and with which significance you see a GO enrichment.
L357 these groupings are probably more true to the data as I expect less bias in transcript mapping than in SNP calling (but potential bias should be addressed here too as per my comments above)
L394-411 see my other comments; I recommend toning down the claims based on the current data.
L401 I wouldn't use the word "evidence". Your result suggest something; but no proof is provided.
L417 more information should be given pertaining to the choice of these genotypes, and not others, among those part of the larger experiment.
L431-433 rephrase this section to make clearer the difference between the N starvation / N treatment design. The text comes across unclear.
L445 this means that leaves were pooled among the two plants making up each replicate as per L435?
L471 I do not understand the choice of the bread wheat genome as a reference, being that you only have tetraploid samples in the dataset and that most if not all have a high quality reference genome available. It is true that to enable comparisons across species it is necessary to use a common reference, but the durum wheat genome would have been much more appropriate, especially due to the nature of the very detailed analyses downstream including variant calling.

Reviewer #2 (Comments for the Author):

Pieri and colleagues collated transcriptomics data from three durum wheat species cultivated on two nitrogen conditions. They used well established approach to identified differently expressed genes, as well as relatively novel Qst-Fst comparison to detect genes under selection during primary and secondary durum wheat domestication. Overall, I think this is timely work given the growing interest in understating the impact of loss of genetic diversity in modern cultivars on agriculture and ability to create new varieties necessary to achieve sustainable development goals. Nevertheless, I have few comments to the paper:

Major Comments:
• The authors previously utilized the Qst-Fst comparison in their earlier work (Beleggia et al. 2016) with metabolomics data, and this approach has been embraced by numerous other researchers. This analytical method has not been widely adopted in the context of transcriptomics analysis so far. Nevertheless, it holds considerable potential for broader application in this domain, especially given the increasing interest in comprehending the influence of selection at the transcriptomic level and the growing availability of relevant data. However, the authors' current approach lacks a comprehensive description, and I believe that their work would greatly benefit from a more detailed discussion of their methodology. For instance, the criteria used for filtering appear to be somewhat arbitrary, and quite strong - for example removing genes with heritability < 0.7 remove substantial portion of genes from the analysis. Is it possible for the Qst-Fst comparison to identify signatures of selection in genes with lower heritability? Under what circumstances can Qst-Fst reveal genes under selection that classical DEG analysis cannot detect, and vice versa? Providing examples to illustrate these points would also enhance the quality of the work.
• The method used for calculating heritability is unconventional. Usually, heritability primarily refers to the proportion of variation linked to genotypes. However, in this case, the authors have incorporated variation associated with species (Vs) into the numerator of the heritability formula (line 545). The rationale behind this inclusion remains unclear. Is there any precedent in the scientific literature for such an approach? If there isn't a compelling justification for this approach, I would recommend considering the exclusion of species variance (Vs) from the numerator and reevaluating the heritabilities of gene expression using only genotype variance (Vg).
• Qst values were computed for six distinct comparisons. However, it appears that the GO analysis was performed for only three of these comparisons, as depicted in Figure S2. It is not clear from the text why the Qst values from two

different nitrogen conditions were combined into a single analysis. I could not find any justification for this in the text.
• The authors assert in the conclusions that they have developed a pipeline for identifying phenotypic plasticity based on the Qst-Fst scores (as mentioned in line 407). However, this connection between Qst-Fst scores and phenotypic plasticity is not sufficiently explained in the main results and discussion section of the paper. Elaborating on this connection will help readers understand the methodology and its implications for identifying phenotypic plasticity more effectively.
• The authors have identified 101 overlapping genes between the DEG and the Qst-Fst approach. However, the presentation and discussion of these results do not include any graphical illustration. It would be beneficial to visually represent the expression patterns of these selected genes in all three species under both nitrogen conditions. This visual representation can help readers better understand and interpret the findings and provide a more comprehensive view of the results.
• Lines 356 - 358: I believe that all the DEGs successfully distinguished the durum wheat genotypes under high nitrogen conditions. On the Principal Component 1 (PC1) axis, it appears that the Lucanica genotype is positioned around ~2, while the closest durum wheat genotype, likely Pedroso, is located at approximately ~8. I agree that on low N there is no clear separation. Speaking of this, how does PCA looks on full set of genes?
• The explanation of how Qst was calculated (line 556 - 557) seems to be truncated. Please clarified this.
• Last but not least: Would be possible to deposit all scripts used in the analysis in one of the relevant repository (such as Zenodo or any other which authors prefer)?

Minor Comments:
• The distribution of Qst on Fig 3b seems to be skew. Are means valuable here (lines 195-197)? Maybe it will more valuable to provide medians in the text?
• Table 1: What is the purpose of introducing theta (θ)? It is not discussed in the text. Also introducing pi (π) as a symbol in the text will increase readability.
• Table S3: In headers of both table there is the same number of genes. Therefore, it is unclear results for which group of genes (DEG and not DEG) are presented in which table.
• Table S4: It will valuable for transparency to add raw Qst values to the table.
• Line 311: the citation are in number format.

Reviewer #3 (Comments for the Author):

Pieri et al. conducted a population genomics analysis using 32 wheat genotypes representing different domestication stages. They investigated gene expression profiles under two nitrogen (N) conditions in four-week-old seedlings and employed Qst-Fst analysis to identify genes under selection. The manuscript is well-written and easy to follow. While the study addresses a crucial topic for agroecosystems and agricultural sustainability, given changing N application practices, some concerns need to be addressed or clarified.

One significant concern relates to potential mapping reference bias. It would be beneficial if the authors highlighted the genetic distance between the reference genome and the three wheat populations. This would help demonstrate that mapping bias should not significantly affect gene expression levels. Given the expected variations in polymorphic sites among wild emmer, emmer, and durum wheat, it is important to address how these differences may impact mapping rates and subsequently affect the validity of the Qst and Fst calculation and differential gene expression analysis.

The concept of "shared SNP" should be defined clearly within the paper. It would be helpful to clarify whether singleton (or private) SNPs within a sub-population are considered segregating sites, or if there are some other criteria. Consider incorporating a site frequency spectrum analysis, which would provide more convincing insights into the distribution of segregating SNP sites.

The nucleotide diversity estimates are based on SNPs from expressed genes at the seedling stage. However, it is unclear whether this accurately reflects the genetic diversity during the two domestication stages. Comparing these estimates with data calculated using genomic data would strengthen the study's findings and provide a more comprehensive understanding of genetic diversity.

The authors might want to include an investigation into the overall gene expression levels under low N conditions and explore any associated phenotypic differences. It is important to consider whether the observed low coefficient of variation (CV) values may be attributed to absolute gene expression levels influenced by N stress.

The GO term enrichment analysis, as presented starting from line 219 and later starting from line 291, appears to be overinterpreted and lacks solid evidence to support the conclusions. A more rigorous analysis and stronger evidence should be provided to substantiate the findings in these sections.

It is essential to clarify whether fold change was considered when determining differentially expressed genes (DEGs).

We thank all three reviewers for providing their feedback on our manuscript. We appreciate the time and effort they have invested in reviewing our work.

We have considered their comments carefully, and we believe that our responses have significantly contributed to strengthening the quality and clarity of our manuscript.

Below, we present a point-by-point response to the reviewers, referring to line numbers based on the post-revision PDF, with highlighted changes.

Thank you once again for your time and consideration.

General response to address reviewers concerns on the choice of reference genome: This topic was a major concern for reviewer #1 and was also raised by reviewer #3, so we group in this first section all the comments and our responses concerning this issue.

Comments related to the reference genome from Reviewer #1:

In this manuscript the authors use a large transcriptomic dataset coupled with experimental work across a range of wheat-realated species to answer very interesting quiestions about ecolution and adaptation. While the manuscript presents an interesting concept and may provide a unique contribution to the field, it seems to me that it suffers from some methodological issues that prevent its publication in its current form. I detail my criticisms below; the authors should take them in consideration and make sure to adjust their claims to what is actually supported by the data. The english language should be improved. Here below, my major concerns; below, line by line comments.

##Major issues:

There are two components in this manuscript: one relative to SNP diversity and one to gene expression.

While interesting and quite innovative, I believe that the former is flawed or at least impaired by underling bias with the data. This all traces back to the reference genome used to call SNPs. Being bread wheat (a choice that I do not agree with, see below), I believe that it is expected that the diversity observed in the dataset follows a gradient going from cultivated (low distance from bread wheat, low diversity) to wilds (high distance from bread wheat, high diversity). This is not to say that this is not generally true, as indeed it is reported by many studies, but rather that it is not supported by the data. As things stand, diversity is not assessed in an even manner; it is biased by looking through the lens of the bread wheat genome. This bias is not desirable nor necessary, since high quality genome sequences are available for the species under study. If the authors wish to report on the SNP diversity, then I suggest supporting the findings by a new alignment and SNP calling for each of the species; once that is done, it is possible to compute measures of nucleotide diversity among individuals of each species.

The second component of the manuscript, that is transcriptomics, is nicer and potentially very relevant, although it can also be improved quite substantially (see more specific comments below). Also in this case, I wonder how the results may be biased by the fact that the authors chose to use bread wheat as reference genome. The explanation given in L471 did not convince me and I would suggest to rather use durum wheat as a reference, if not multiple references as required by my point above.

L93 provide data specific for each species (see comment about methods)

L97-100 although it is certainly true that DW diversity is lower than that of wilds, I think that this is not necessarily the only explanation as your SNP data will be significantly biased towards cultivated wheat. Simply, DW is more similar to BW that you are using as a reference and hence you detect less SNPs. I expect a similar pattern in mapping % (see comment above)

L103-106 same as above; this claim, although possibly true, results from a biased dataset. If ones would like to compare the amount of diversity available in each of the three species, assuming that the selection of genotypes is representative of the species, this should be done on SNPs called on the respective reference genomes.

L115 see comment above

L471 I do not understand the choice of the bread wheat genome as a reference, being that you only have tetraploid samples in the dataset and that most if not all have a high quality reference genome available. It is true that to enable comparisons across species it is necessary to use a common reference, but the durum wheat genome would have been much more appropriate, especially due to the nature of the very detailed analyses downstream including variant calling.

Comment related to the reference genome from Reviewer #3:

One significant concern relates to potential mapping reference bias. It would be beneficial if the authors highlighted the genetic distance between the reference genome and the three wheat populations. This would help demonstrate that mapping bias should not significantly affect gene expression levels. Given the expected variations in polymorphic sites among wild emmer, emmer, and durum wheat, it is important to address how these differences may impact mapping rates and subsequently affect the validity of the Qst and Fst calculation and differential gene expression analysis.

Response: We acknowledge that selecting the appropriate reference genome for our analysis was a critical decision, given the presence of genotypes from three different subspecies. This posed challenges in terms of comparing results among groups, including nucleotide variants and gene expression profiles.

Initially, considering the availability of reference genomes for only two of the three subspecies in our study (wild emmer and durum wheat), we chose to use *Triticum aestivum* (bread wheat) A and B subgenomes as an outgroup reference, given its

close relationship to the tetraploid wheats included in our study. This decision aimed to prevent potential biases that could arise from favoring one subspecies over the others. While this decision was made after extensive deliberation and consultation, we acknowledge that the choice of reference genome may still influence certain aspects of our analysis.

Therefore, following the reviewer suggestions, we reproduced our analysis using the reference genomes of wild emmer (*T. turgidum* ssp. *dicoccoides* Zavitan) and durum wheat (*T. turgidum* ssp. *durum* Svevo). By adopting this approach, we aimed to address concerns regarding the choice of reference genome and ensure the robustness and validity of our findings.

In detail, we repeated:

- read alignments
- transcript quantifications
- SNP calling
- PCA
- nucleotide diversity estimates

We have incorporated the outcomes of these additional analyses in our revised manuscript by adding a dedicated Supplemental Methods 1 text file, which describes in detail the validation of the choice of the reference genome. Moreover, the full statistical analysis of the new alignments is included in Supplemental Data Set S1, we added a new Supplemental Data Set S2 reporting the number of SNPs and nucleotide diversity estimates for each reference genome, and we added a new Supplemental Figure S2 with PCA performed using SNPs called from the wild emmer and durum wheat genomes.

Here is a summary of the results:

- <u>Read alignments and transcript quantifications</u>: Mapping frequency to the whole genome was comparable among the three references, ranging from 86% to 87%. This suggests that the reads align well across the genomes. A similar fraction of reads (~73%) mapped to gene regions in bread wheat and wild emmer, while a lower fraction (~52%) was observed in durum wheat. This decrease is attributed to the lack of untranslated regions (UTRs) in the Svevo reference annotation, negatively impacting transcript quantification. Indeed, this resulted in a lower number of genes detected using the Svevo reference genome (Supplemental Methods 1).

  In response to reviewer #3, we also calculated the genetic distances and highlighted the >98% average nucleotide identity between the bread wheat Chinese Spring A and B subgenomes, durum wheat Svevo genome, and wild emmer Zavitan genome, confirming their close relationship (Supplemental Methods 1). A ~98% average nucleotide identity was also observed in the three gene sets, obtained using the three different references.

- <u>SNP calling, PCA, nucleotide diversity</u>:

The use of wild emmer and durum wheat references reduced the number of SNPs compared to the bread wheat reference, with 604,479 SNPs identified using wild emmer 544,406 using durum wheat, and 800,996 SNPs using bread wheat (Supplemental Methods 1). However, the similarity in the number of polymorphic sites between wild emmer and emmer, alongside the lower number of durum wheat SNPs, was reaffirmed using both alternative references, as well as the ratio of private and shared SNPs (Supplemental Data Set S2). The equivalence of the three references was further evidenced by identical PCA results derived from the SNPs called using wild emmer and durum wheat (Supplemental Figure S2).

Additionally, π and θ estimates showed lower subspecies-dependent values compared to those obtained using the bread wheat reference (Supplemental Data Set S2). The π values obtained using the wild emmer reference were 0.0027 for wild emmer, 0.0027 for emmer, and 0.0024 for durum, whereas those obtained using the durum wheat reference yielded similar but notably lower values, especially in durum wheat (π = 0.0011). The overall trend of diversity loss confirmed previous findings using the bread wheat reference, indicating secondary domestication had a more pronounced impact. The percentage losses of π nucleotide diversity observed using the *T. aestivum* reference (11.4% loss during primary domestication and 16.8% during secondary domestication) fell between those obtained using wild emmer (3.2% loss during primary domestication and 10.1% during secondary domestication) and durum wheat (12.5% loss during primary domestication and 48.1% during secondary domestication).

Therefore, despite minor differences in the outcomes across different references, the overall patterns remain consistent and the use of an outgroup species for reference might help mitigate biases and ensure fair representation of all subspecies, maintaining mapping accuracy and coverage of gene regions, making it a suitable choice for our analysis.

Reviewer #1:

##Other general and important comments:

This is an interesting experimental setup, but I wonder to what extent N availability can be put in relation to domestication (primary or secondary). Is there any information about use of fertilizers in association with early farming and how this aligns with the timeline of domestication? It seems a bit stretched to put in relation N availability to genomic signatures of selection across domestication, unless more context is provided to justify this hypotesis.

Response: We added to the introduction (line 105 et seq) to provide more context for the relationship between N availability and domestication. We thank the reviewer for helping to clarify the link between N utilization and domestication.

If I got it right, variants were called on transcriptomic data. Authors need to discuss the implications of this approach, especially since these variants come from alignment to a species not included in the dataset. Are these variants causative of the adaptation or rather they are in linkage with upstream or downstream variants which may play a role? This should be acknowledged. If SNP calling gets improved as per my suggestions above, it would be quite relevant to look for synonymous/non synonymous exonic variants in the dataset to further support the interpretation of evolutionary processes in relation to N use. This would enrich the GO analysis that, as it stands, is quite general and not very convincing.

Response: In the way we conceived our methodology, we did not expect to detect the variants causative of the adaptation. Instead, we focused on developing a "selection scan" approach, through which we identified genes that showed significant differences in expression levels (high Q_{ST} values), but relatively minor differences at the nucleotide level (lower F_{ST} values) (as also clarified in the text, line 299). This suggests that the signals we observed likely stem from regulatory mechanisms affecting gene expression, rather than mutations within the gene's coding regions that could alter gene products. In response to reviewer's suggestion, we further analyzed the presence of non-synonymous and synonymous variants in the genes identified as under selection, comparing them with an equal number of randomly selected genes from our dataset. We discovered a notable decrease in both types of variants in the genes under selection, particularly in durum wheat. This supports our hypothesis that these candidate genes, potentially under directional selection, might have also undergone purifying selection. We revised our manuscript by adding a Supplemental Methods 2 text file dedicated to this analysis.

##Line by line comments

L31. Genotypes or species? Say a few words to specify this.

L33. Refrain from using jargon in abstract. A naïve reader may not know what Qst is. Same for primary and secondary domestication.

L36 Nitrogen what? Quantity? Availability?

Response: We have rephrased the abstract in order to make it clearer and easier to read.

L66-67 it seems that there is a logic jump over here. How phenotypic plasticity relates to the previous paragraph? Rephrase.

*L69 this sentence reads a bit controversial; I believe that SDGs are aligned with a *reduction* of fertilizer use.*

Response: The introduction has been revised as highlighted in the text (from line 105), following these suggestions.

L137 I am not sure what is the meaning of "nucleotide diversity in this context"

L141 this is a very interesting result, but I don't see how it relates to the association between domestication and diversity reported in L142-144

Response: We have rephrased this section, as highlighted (from line 208).

L146 interesting plots; perhaps a log scale would allow to see differences between species better?

Response: We think that maintaining the same CV_A values, as reported in the corresponding Table 2, helps the reader to follow the discussion and link the plots with the interpretation.

L183 VERY strong claim, given the limitations discussed above and below. I suggest to tone down a bit here and elsewhere.

Response: We have changed the title to tone down our claim.

L190-191 I would argue that this is still no proof of "representativeness" of the collection. I suggest discussing this aspect in more detail, noting that a limitation of the study is the limited number of genotypes considered (although for good reasons that I fully understand).

Response: We have rephrased this sentence (line 256) and mentioned the limitation of our number of samples (line 518).

L223-267 This are a nice stories but I wonder if the data really support an interpretation going this far. It seems quite speculative given the type of data and genetic materials; I would reduce this part and note it as speculation.

Response: We acknowledge the speculative nature of some interpretations. However, this section is relevant so we have revised it to reduce speculation and clearly delineate where interpretations are based on more tentative grounds. Moreover, to address a comment raised by reviewer #2, concerning the GO analysis performed only on three groups of genes, we repeated the analysis keeping the six groups separate, and we modified the text accordingly (from line 313 and Supplemental Figure S5).

L305 reference formatting is odd here

Response: The formatting has been corrected.

L309 provide more details about which genes and how many, and with which significance you see a GO enrichment.

Response: We have provided a detailed report of the GO enrichment in Supplemental Data Set S5, along with the functional annotation of the most strongly modulated genes in Supplemental Data Set S6. Considering the nature of our study, we believe that these analyses are sufficiently comprehensive. Our intention was not to delve too deeply into individual DEGs because our work does not represent a "classical" transcriptomics analysis of wheat in response to contrasting nitrogen conditions, for which we have cited previous studies (line 406 et seq).

L357 these groupings are probably more true to the data as I expect less bias in transcript mapping than in SNP calling (but potential bias should be addressed here too as per my comments above)

Response: As described in the first response, we show how the PCA groupings of SNPs reproduced using different reference genomes are entirely comparable to those obtained using bread wheat.

L394-411 see my other comments; I recommend toning down the claims based on the current data.

L401 I wouldn't use the word "evidence". Your result suggest something; but no proof is provided.

Response: We have reworded the last section as recommended by the reviewer.

L417 more information should be given pertaining to the choice of these genotypes, and not others, among those part of the larger experiment.

Response: We added an explanation (lines 536-539).

L431-433 rephrase this section to make clearer the difference between the N starvation / N treatment design. The text comes across unclear.

Response: We have rephrased as the text (lines 550-555).

L445 this means that leaves were pooled among the two plants making up each replicate as per L435?

Response: Yes, this is correct.

Reviewer #2:

Pieri and colleagues collated transcriptomics data from three durum wheat species cultivated on two nitrogen conditions. They used well established approach to identified differently expressed genes, as well as relatively novel Qst-Fst comparison to detect genes under selection during primary and secondary durum wheat domestication. Overall, I think this is timely work given the growing interest in understating the impact of loss of genetic diversity in modern cultivars on agriculture and ability to create new varieties necessary to achieve sustainable development goals. Nevertheless, I have few comments to the paper:

Major Comments:

• *The authors previously utilized the Qst-Fst comparison in their earlier work (Beleggia et al. 2016) with metabolomics data, and this approach has been embraced by numerous other researchers. This analytical method has not been widely adopted in the context of transcriptomics analysis so far. Nevertheless, it holds considerable potential for broader application in this domain, especially given the increasing interest in comprehending the influence of selection at the transcriptomic level and the growing availability of relevant data. However, the authors' current approach lacks a comprehensive description, and I believe that their work would greatly benefit from a more detailed discussion of their methodology. For instance, the criteria used for filtering appear to be somewhat arbitrary, and quite strong - for example removing genes with heritability < 0.7 remove substantial portion of genes from the analysis. Is it possible for the Qst-Fst comparison to identify signatures of selection in genes with lower heritability? Under what circumstances can Qst-Fst reveal genes under selection that classical DEG analysis cannot detect, and vice versa? Providing examples to illustrate these points would also enhance the quality of the work.*

• *The authors assert in the conclusions that they have developed a pipeline for identifying phenotypic plasticity based on the Qst-Fst scores (as mentioned in line 407). However, this connection between Qst-Fst scores and phenotypic plasticity is not sufficiently explained in the main results and discussion section of the paper. Elaborating on this connection will help readers understand the methodology and its implications for identifying phenotypic plasticity more effectively.*

Response: We agree with the reviewer that the discussion of the method was not as clear or as valorized as it could have been. We have now introduced a paragraph to outline our methodology and its link with phenotypic plasticity (from line 268). Additionally, we have attempted to clarify the connection we made between the Q_{ST}-F_{ST} methodology and the "classical" DE analysis (from line 478). Our goal was to show how we integrated these two independent analyses to support our results.

We specify that, with our promising results, we aimed to provide proof of concept for our methodology, which can be further improved and adjusted (e.g., by choosing less

stringent thresholds when increasing statistical power with larger number of genotypes).

We believe that these additions have significantly enhanced the quality and comprehensibility of our work and we thank the reviewer for raising this issue.

• *The method used for calculating heritability is unconventional. Usually, heritability primarily refers to the proportion of variation linked to genotypes. However, in this case, the authors have incorporated variation associated with species (Vs) into the numerator of the heritability formula (line 545). The rationale behind this inclusion remains unclear. Is there any precedent in the scientific literature for such an approach? If there isn't a compelling justification for this approach, I would recommend considering the exclusion of species variance (Vs) from the numerator and reevaluating the heritabilities of gene expression using only genotype variance (Vg).*

Response: We understand the concerns raised by the reviewer and acknowledge the departure from the traditional approach, where heritability primarily reflects the proportion of variation attributed to genotypes. The rationale behind our method is in the nature of our study, which focuses on a characteristic panel of accessions grouped into three subspecies. In this context, our primary interest lies not in analyzing individual genotypes, but rather in examining the three subspecies as distinct groups, with the genotype factor being nested within the species. Therefore, in all our analyses, we have presented results pertaining to these three groups. We believe this approach provides valuable insights for understanding the genetic variation within these subspecies groups.

• *Qst values were computed for six distinct comparisons. However, it appears that the GO analysis was performed for only three of these comparisons, as depicted in Figure S2. It is not clear from the text why the Qst values from two different nitrogen conditions were combined into a single analysis. I could not find any justification for this in the text.*

Response: The reviewer is correct. Initially, we combined the two N conditions into a single analysis, in order to recover more genes in each group to increase statistical power, and a justification in the text was missing. We therefore decided to repeat the analysis keeping the six groups separated and we modified the text accordingly (from line 313 and Supplemental Figure S5). We believe that the new GO analysis reinforces our claims, specifically by highlighting how the categories previously identified for primary and secondary domestication were solely detected under high-N conditions, thereby reinforcing the relevance of our results in the context of N availability.

• *The authors have identified 101 overlapping genes between the DEG and the Qst-Fst approach. However, the presentation and discussion of these results do not*

include any graphical illustration. It would be beneficial to visually represent the expression patterns of these selected genes in all three species under both nitrogen conditions. This visual representation can help readers better understand and interpret the findings and provide a more comprehensive view of the results.

Response: We have added a heat map of the expression patterns as Supplemental Figure S9.

• *Lines 356 - 358: I believe that all the DEGs successfully distinguished the durum wheat genotypes under high nitrogen conditions. On the Principal Component 1 (PC1) axis, it appears that the Lucanica genotype is positioned around ~2, while the closest durum wheat genotype, likely Pedroso, is located at approximately ~8. I agree that on low N there is no clear separation. Speaking of this, how does PCA looks on full set of genes?*

Response: Initially, we included PCA on the entire set of genes in the panel alongside the other two PCAs. However, we later decided to remove it because we felt it was burdening the discussion. We have now reintroduced PCA on the entire gene set as Supplementary Figure S10.

• *The explanation of how Qst was calculated (line 556 - 557) seems to be truncated. Please clarified this.*

Response: We have clarified this issue (line 689).

• *Last but not least: Would be possible to deposit all scripts used in the analysis in one of the relevant repository (such as Zenodo or any other which authors prefer)?*

Response: The scripts used in the study are now available at GitHub, as specified in the text (line 794).

Minor Comments:

• *The distribution of Qst on Fig 3b seems to be skew. Are means valuable here (lines 195-197)? Maybe it will more valuable to provide medians in the text?*

Response: We agree and have now provided medians instead of means for both F_{ST} and Q_{ST} (lines 252 and 261).

• *Table 1: What is the purpose of introducing theta (θ)? It is not discussed in the text. Also introducing pi (π) as a symbol in the text will increase readability.*

Response: We have revised this section as requested to improve readability.

• Table S3: In headers of both table there is the same number of genes. Therefore, it is unclear results for which group of genes (DEG and not DEG) are presented in which table.

Response: We have corrected the error in the table, which is now Supplemental Table S1.

• Table S4: It will valuable for transparency to add raw Qst values to the table.

Response: We agree and have added Q_{ST} values to the table, which is now Supplemental Data Set S4.

• Line 311: the citation are in number format.

Response: We have reformatted the text accordingly.

Reviewer #3:

Pieri et al. conducted a population genomics analysis using 32 wheat genotypes representing different domestication stages. They investigated gene expression profiles under two nitrogen (N) conditions in four-week-old seedlings and employed Qst-Fst analysis to identify genes under selection. The manuscript is well-written and easy to follow. While the study addresses a crucial topic for agroecosystems and agricultural sustainability, given changing N application practices, some concerns need to be addressed or clarified.

The concept of "shared SNP" should be defined clearly within the paper. It would be helpful to clarify whether singleton (or private) SNPs within a sub-population are considered segregating sites, or if there are some other criteria. Consider incorporating a site frequency spectrum analysis, which would provide more convincing insights into the distribution of segregating SNP sites.

Response: We have revised the results section that covers variant calling from line 167, and have included a clarification addressing the concerns raised by the reviewer and a site frequency spectrum analysis as Supplemental Figure S1.

The nucleotide diversity estimates are based on SNPs from expressed genes at the seedling stage. However, it is unclear whether this accurately reflects the genetic diversity during the two domestication stages. Comparing these estimates with data calculated using genomic data would strengthen the study's findings and provide a more comprehensive understanding of genetic diversity.

Response: We acknowledge the limitations of this approach, which primarily considers expressed regions of the genome. The incorporation of genomic data is outside the scope of the reported study which aimed to leverage transcriptomic data to investigate nucleotide diversity and selection signatures beyond conventional differential expression analysis. Nevertheless, throughout the manuscript, we emphasize the alignment of our findings with other studies across various species, and with earlier investigations that analyzed extensive collections of tetraploid wheat genomic data.

The authors might want to include an investigation into the overall gene expression levels under low N conditions and explore any associated phenotypic differences. It is important to consider whether the observed low coefficient of variation (CV) values may be attributed to absolute gene expression levels influenced by N stress.

Response: An extensive study conducted by Gioia et al. (2015) investigated the phenotypic differences associated with different N conditions, which also included our genotypes. Indeed, we value those findings and link them with our observations on the CV_A, (line 237 et seq). Although N stress is expected to influence gene expression levels, we do not see how it could negatively affect the estimation of the CV_A which has the scope of comparing the gene expression diversity among the subspecies, exactly in the two different N conditions. Moreover, we observe overall higher mean CV_A values in low-N compared to high-N conditions, suggesting higher variability in N stress, as explained in the revised text (line 208 et seq).

The GO term enrichment analysis, as presented starting from line 219 and later starting from line 291, appears to be overinterpreted and lacks solid evidence to support the conclusions. A more rigorous analysis and stronger evidence should be provided to substantiate the findings in these sections.

Response: We understand these concerns regarding the speculative nature of some interpretations in the GO term enrichment analysis. We have therefore repeated the analysis while keeping separate the six groups of genes with selection signatures, as also mentioned in our response to reviewer #2. This revision is reported in the revised text (from line 313 and Supplemental Figure S5).

We believe that the new GO analysis strengthens our claims, particularly by highlighting how the categories previously identified for primary and secondary domestication were solely detected under high-N conditions. This reinforces the relevance of our results in the context of N availability. We have revised this section to reduce the amount of speculation.

We have also provided a detailed report of the GO enrichment categories relevant to DEGs in Supplemental Data Set S5, along with the functional annotation of the most strongly modulated genes in Supplemental Data Set S6.

A detailed functional annotation of all the genes is beyond the scope of our investigation and we believe that the reported analyses are sufficiently comprehensive to support our conclusions.

It is essential to clarify whether fold change was considered when determining differentially expressed genes (DEGs).

Response: When presenting the results in terms of numbers of DEGs, we did not filter for any fold change threshold. We opted for this approach because, after applying a stringent pipeline and strict thresholds ($p_{adj} < 0.001$) to reduce the number of false positives (line 702 et seq), we believe that each gene, even if not strongly modulated, should be considered informative. However, we understand the importance of focusing on genes with stronger modulation. Therefore, we have provided functional annotations for the most strongly modulated genes in Supplemental Data Set S6. This allows a more in-depth examination of the genes with the most significant expression changes while still acknowledging the informative nature of all DEGs in our study.

Prof. Roberto Papa
Department of Agricultural, Food and Environmental Sciences
Marche Polytechnic University
Ancona 60131
Italy

Dear Roberto / Dr. Papa:

We have received reviews of your manuscript entitled "Transcriptomic Response to Nitrogen Availability Highlights Signatures of Adaptive Plasticity During Tetraploid Wheat Domestication." Thank you for submitting your best work to The Plant Cell. The editorial board agrees that the work you describe is substantive, falls within the scope of the journal, and may become acceptable for publication, pending revision and potential re-review.

We ask you to pay attention to the following points in preparing your revision:

1) Removal of results and discussion from supplementary methods.
2) Address the question of the correct background set to use in GO analysis.
3) Address reviewer #3's ongoing concerns regarding the integration of the new analyses with different reference genomes more fully into the revised manuscript.

Please contact us if there are ambiguous comments or if you wish to discuss the revision.

Given the nature of the comments, we are offering you 60 days from when we have issued this decision to complete the revision. If a revision is not returned within this time frame, and if you have not been granted an extension, we will withdraw the manuscript, which will leave you free to submit the work elsewhere. If you need an extension, we encourage you to contact us at any point before the 60 days have passed.

When you are ready to submit the revised version, please upload a highlighted copy that indicates all changes made in response to the editor and reviewer recommendations. Include an itemized list of all changes made in response to each of the reviewer's suggestions in the "Response to Reviewers" section; please note that reviewers do not have access to your cover letter, nor was this decision letter shared with them.

Thank you for the privilege of reviewing your work. We look forward to receiving your revised manuscript.

Sincerely,

The Plant Cell Board of Editors

Please note the following:

-The Plant Cell now requires authors to complete and submit an author revisions checklist upon submission of a revised manuscript. The aim of the checklist is to aid authors in preparing a high-quality manuscript, facilitate the review and assessment of revised manuscripts, and help to ensure that journal standards are maintained across the board. If your manuscript is accepted, the completed checklist will be published as supplemental material attached to the article online. Please download a copy of the checklist (pdf fillable form) at this link, for submission with your revised manuscript: https://tpc.msubmit.net/html/Author_Revisions_Checklist.pdf.

-Supplemental materials should be restricted to large datasets and tables, presentation of replicates, and validation of reagents, methods, or genotypes. Any data that are used to support the major claims must be in the main manuscript. Supplemental figure legends must indicate what figure in the main manuscript is supported by the supplemental data presented. Please justify how each of the supplemental figures meet the criteria.

-Sampling methods and nature of "biological replicates" should be described precisely (i.e. different plants, parts of plants, pooled tissue, independent pools of tissue, sampled at different times, etc), along with a clear description of and rationale for any statistical analyses conducted. The reader should know exactly what was sampled; what forms the basis of the calculation of any means and statistical parameters reported. This is also necessary to ensure that proper statistical analysis was conducted.

-Want to add this revision deadline to your calendar? Click below!

-- Reviewer comments:

Reviewer #1 (Comments for the Author):

The authors did a good job in addressing the requests for revisions; I appreciate the addition of analyses and results that clarified the most critical points. Although the manuscript has markedly improved in its content, I believe that there is still work to be done on its presentation. The text is too long, at times repetitive, and in several occurrences claims are too bold when considering the limitations of the data available. The manuscript text can be tidied and made much more effective with some more work. Especially the revised sections (highlighted in yellow) have somewhat poorer language editing. Figures are informative appropriate. The supplementary methods sections are informative, but they contain also results and discussion and I don't think that this is appropriate. All in all, I would be happy to see this published after a few more edits and text revisions.

Point by point comments below:

L33 I suggest revising English language for correctness and quality. This sentence sounds odd and not particularly informative. What does "agroecosystem development" mean in this context? Which "environmental changes" occurred? Unclear
L36 English revision; I don't think these species can be called "stages". Perhaps "steps"? Moreover one of the species IS tetraploid wheat.
L40 is this during primary domestication or across primary domestication?
L55 also here not clear what you mean for "development of agricultural ecosystems"
L63 italics missing
L106 rephrase; the overreliance is true for modern farming but not for early farming (as the first sentence suggests). I think that all the revised section of the document would benefit for a thorough revision of the English language
L106-118 I think this all section is appropriate and informative, but it should be revised and reorganized; first discussing early farming, then modern farming. Right now it reads Donald model, then neolithic, then Haber-Bosch and it's a bit confusing
L128 I don't think that phenotypic plasticity is necessarily related to transition from wild to farming; we are talking about evolutionary processes lasting thousands of years. I agree in the importance of introducing phenotypic plasticity and putting it in relation with RNA seq, but the section should be rephrased.
L138-142 it doesn't seem to me that this is the best description of your work. You looked at RNA expression in relation to N availability using species representing wheat domestication steps to shed light on species-specific response to N and hence on N role during domestication. You did NOT "determine how N availability shaped nucleotide and gene expression diversity", at least not experimentally.
L149 you may want to add a couple of words about the experimental conditions in which the plants were grown.
L156 I don't think that "intermediate" is the most appropriate word.
L161 I approve this revision but I suggest rephrasing and improving language in the paragraph. The information given in L161-164 should be given before L150.
L167 clarify that this is variant calling on RNA seq.
L174 for consistency, call them species rather than taxa (here and elsewhere in the text)
L195-198 I approve this revision but the text is not very clear. The paragraph can be rephrased for clarity. "To ensure that our results were not biased towards the chosen reference genome, we [...]
L203 here you introduce "high and low N availability conditions" but it is not clear how these have been tested on the genoypes and how these related to the RNA seq libraries
L209 Rephrase. "CV was higher in wild emmer, lower in emmer, and lowest in durum wheat". The next sentence: "The loss of diversity in gene expression has been observed across the domestication process in other crops"
L243 "may have favoured"
L289-304 I understand the rationale for this stringent filtering and I support it. However, I wonder whether this stringency may have left out genes important for the trait under study (N availability). The overall picture is certainly solid, but could it be that the number of genes retained (less than 18%) is so small that it is not representative when looking at candidate functions and GO? I'd like this to be discussed
L318 enriched in wild wheat or cultivated wheat?
L321 Still I am not convinced that N availability could or should be considered this overwhelming selection pressure in relation to domestication. It is OK to discuss how different wheat species representing the domestication process of wheat react differently to N availability. I don't think it is OK to treat N availability as a cause of this differentiation (in evolutionary terms) as it is suggested by this sentence and elsewhere.
L324-355 this section is largely speculative, and I suggest reducing it substantially. The whole discussion about pathogen resistance, or lysine, SACPATH etc, are potentially interesting, but are not supported by experimental

evidence sufficient to warrant such a detailed discussion. I think that authors should make a further effort to avoid chasing those results that support their hypothesis, but rather stick to the description of the findings (that are already interesting enough without the need of speculation)
L326 here and elsewhere, whenever "enrichment" is reported, add p values
L522 remove "pioneering" and all claims about the exceptionality of this study. It is up to the reader to assess this.
L527 is this sentence missing a verb?
L794 this is nice, and reinforces the methodological ambition of this manuscript

Reviewer #2 (Comments for the Author):

In the resubmitted paper, the authors analyzed the impact of domestication on transcriptome plasticity under nitrogen starvation in durum wheat. The most significant change in the article is the additional analysis, which demonstrates the selection of the bread wheat genome as the optimal reference for the analysis. Overall, I believe that my queries have been addressed as well.
I have two additional question:

The authors applied several arbitrary filters to the gene expression data prior to the QST-FST analysis, reducing the number of analyzed genes to 5,868. However, the population background used in the gene ontology analysis is not specified. I assume that all annotated wheat genes were used as the background population. It is possible that the categories found to be over represented in the 967 genes that satisfied the QST-FST analysis criteria are already over represented in the 5,868 analyzed genes. Can the authors specify the population background used for the analysis? This information could have a significant impact on the results.

Several values of Qst in Data S4 have value 0 or 1. How this can be interpreted?

Reviewer #3 (Comments for the Author):

I appreciate that the authors considered my and other reviewers' suggestions and comments in the manuscript revision, especially their efforts in reanalyzing using wild emmer and durum wheat as reference genomes. However, I feel the re-analyses have not been fully incorporated into the revised manuscript beyond some verbal justification. For example, in the revised manuscript, lines 153-166, the authors claimed that they had corrected the reference bias by mapping reads to tetraploid wheat reference genomes, but they failed to mention any numerical or statistical evidence to support why "bread wheat was the best choice" other than citing the Supplemental datasets. Also, these supplemental datasets should be visualized either as main figures or supplementary figures. Otherwise, it is hard to understand why the reference bias wouldn't affect the interpretation of the results.
I expect that genetic distances (such as using the IBS approach) will be calculated pairwise and used to justify why a certain reference would be biased and how to interpret the results accordingly. I do not completely follow what the Mash software is doing and why they calculate the average nucleotide identity. What this average nucleotide identity means is not clearly explained.
In lines 171-174, the authors simply conducted site frequency spectra analysis without any interpretation. I would expect further statistical analysis or comparison by partitioning the SFS into genomic features, such as 5'UTR, exonic, or intronic regions, etc.
When addressing my comments regarding 'private' or 'shared' SNPs, the authors provided a definition as requested. However, I believe there is an opportunity here for a more in-depth analysis. A deeper exploration of 'private' SNPs, if not due to SNP calling error, could potentially reveal the relationship between selection and genetic drift. As hypothesized by Beissinger et al. 2016 in a maize study, low-frequency (such as singleton) or younger polymorphisms might show distinct patterns from pairwise diversity, which is mainly determined by intermediate frequency or older alleles. A comprehensive analysis and comparison with what has been discussed in maize and other species could significantly enhance the paper's strength.

We thank all three reviewers for the additional time and effort they have invested in the second revision of our work. By addressing their additional comments, the claims in the updated manuscript are further streamlined and strengthened.

Below, we present a point-by-point response to the reviewers, referring to line numbers based on the post-revision PDF, with highlighted changes.

Reviewer #1:

The authors did a good job in addressing the requests for revisions; I appreciate the addition of analyses and results that clarified the most critical points. Although the manuscript has markedly improved in its content, I believe that there is still work to be done on its presentation. The text is too long, at times repetitive, and in several occurrences claims are too bold when considering the limitations of the data available. The manuscript text can be tidied and made much more effective with some more work. Especially the revised sections (highlighted in yellow) have somewhat poorer language editing. Figures are informative appropriate. The supplementary methods sections are informative, but they contain also results and discussion and I don't think that this is appropriate. All in all, I would be happy to see this published after a few more edits and text revisions.

Point by point comments below:

L33 I suggest revising English language for correctness and quality. This sentence sounds odd and not particularly informative. What does "agroecosystem development" mean in this context? Which "environmental changes" occurred? Unclear

Response: “Agroecosystem development” refers to the creation and refinement of agricultural systems driven by human needs, a process integral to domestication. This transition from wild environments to early farming systems and eventually to modern agroecosystems has resulted in significant changes in plant growing conditions. These changes are characterized by human intervention and management practices, such as: soil preparation, sowing density, irrigation, fertilization, pest control, and crop rotation. The development of these ecosystems offers a unique opportunity to study plant adaptation because they represent dynamic and controlled settings where both natural and artificial selection pressures are at operation.

We believe that within the context of the abstract, this sentence serves as a background introduction that does not require further explanation, which is provided in the Introduction of the main text.

L36 English revision; I don't think these species can be called "stages". Perhaps "steps"? Moreover one of the species IS tetraploid wheat.

Response: We have revised this sentence to make it clear that the term "stages" pertains to the phases of domestication rather than directly to the subspecies themselves, and that the subspecies are representative of those phases.

L40 is this during primary domestication or across primary domestication?

Response: We agree that the term “across” better defines the transition from one subspecies to another, and we changed the text accordingly.

L55 also here not clear what you mean for "development of agricultural ecosystems"

Response: Here, "development of agricultural ecosystems" has the same meaning as in line 33. Since the "In a Nutshell" section is aimed at a “college-level audience of science enthusiasts rather than scientists”, we believe the meaning is clarified by the preceding sentence: "The process of crop domestication, which involves humans selecting and breeding plants for agriculture, has led to significant changes in the environment where these crops grow”.

L63 italics missing

Response: The formatting has been corrected.

L106 rephrase; the overreliance is true for modern farming but not for early farming (as the first sentence suggests). I think that all the revised section of the document would benefit for a thorough revision of the English language

Response: In response to the next comment, we have rephrased the entire section and also this sentence is now clarified.

L106-118 I think this all section is appropriate and informative, but it should be revised and reorganized; first discussing early farming, then modern farming. Right now it reads Donald model, then neolithic, then Haber-Bosch and it's a bit confusing

Response: We reorganized this paragraph (lines 104-116), and we agree that the readability is now improved.

L128 I don't think that phenotypic plasticity is necessarily related to transition from wild to farming; we are talking about evolutionary processes lasting thousands of years. I agree in the importance of introducing phenotypic plasticity and putting it in relation with RNA seq, but the section should be rephrased.

Response: We have rephrased this sentence (lines 127-128) to better introduce the relationship between phenotypic plasticity and changing environments.

L138-142 it doesn't seem to me that this is the best description of your work. You looked at RNA expression in relation to N availability using species representing wheat domestication steps to shed light on species-specific response to N and hence on N role during domestication. You did NOT "determine how N availability shaped nucleotide and gene expression diversity", at least not experimentally.

Response: We have reworded this section (lines 138-144), and we agree that the streamlining of this section improved description of our work.

L149 you may want to add a couple of words about the experimental conditions in which the plants were grown.

Response: We added the experimental growing conditions (line 168).

L156 I don't think that "intermediate" is the most appropriate word.

Response: We believe that the term "intermediate" effectively addresses the suitability of bread wheat's A and B subgenomes for facilitating comparisons among the different subspecies.

L161 I approve this revision but I suggest rephrasing and improving language in the paragraph. The information given in L161-164 should be given before L150.

Response: We have now incorporated a dedicated paragraph about the choice of the reference genome to the main text (lines 147-199).

L167 clarify that this is variant calling on RNA seq.

Response: We added this information in the text (line 203).

L174 for consistency, call them species rather than taxa (here and elsewhere in the text)

Response: We used the term "*taxa*" throughout the manuscript to refer to the groups of individuals forming a unit (wild emmer, emmer, and durum wheat). This choice helps us avoid repeating "subspecies" excessively.

L195-198 I approve this revision but the text is not very clear. The paragraph can be rephrased for clarity. "To ensure that our results were not biased towards the chosen reference genome, we [...]

Response: We agree with reviewer's suggestion and have rephrased the sentence. In addition, we moved the additional results that were reported as separate Supplemental Methods to the main text.

L203 here you introduce "high and low N availability conditions" but it is not clear how these have been tested on the genoypes and how these related to the RNA seq libraries

Response: We believe that the methodology details are adequately provided in the Materials and Methods section (lines 729-737). Here, we introduce the context of "high and low N availability conditions" and specify that CV_A was calculated based on read counts in the following sentence.

L209 Rephrase. "CV was higher in wild emmer, lower in emmer, and lowest in durum wheat". The next sentence: "The loss of diversity in gene expression has been observed across the domestication process in other crops"

Response: We revised the text according to reviewer's suggestions.

L243 "may have favoured"

Response: We revised the text in line with the reviewer's suggestion.

L289-304 I understand the rationale for this stringent filtering and I support it. However, I wonder whether this stringency may have left out genes important for the trait under study (N availability). The overall picture is certainly solid, but could it be that the number of genes retained (less than 18%) is so small that it is not representative when looking at candidate functions and GO? I'd like this to be discussed

Response: We appreciate the reviewer's support for our filtering choice. As specified in the main text, our methodology was designed as a proof of concept, with the understanding that future experiments may refine and adjust these criteria. The significant GO results and functional annotation highlighted specific gene categories that are in accord with the evolutionary contexts examined, providing a robust foundation for further investigation. While our analysis may not have captured the entire set of genes relevant to N availability, we believe the identified categories are representative and provide a valuable starting point. It is important to acknowledge that every experiment is inherently limited by its specific conditions and should serve as a basis for further research.

L318 enriched in wild wheat or cultivated wheat?

Response: We have revised the sentence (lines 395-397) to clarify that the enrichment was observed in the genes showing selection signatures when comparing wild emmer and durum wheat under low-N conditions.

L321 Still I am not convinced that N availability could or should be considered this overwhelming selection pressure in relation to domestication. It is OK to discuss how different wheat species representing the domestication process of wheat react differently to N availability. I don't think it is OK to treat N availability as a cause of this differentiation (in evolutionary terms) as it is suggested by this sentence and elsewhere.

Response: While we appreciate reviewer's perspective, our intention is not to imply that N availability is the sole or overwhelming selection pressure in relation to domestication. Rather, we aim to explore how N availability might have influenced the evolutionary dynamics of different wheat species during the domestication process. We acknowledge the complexity of domestication and the multitude of factors involved. Our discussion is intended to contribute to a broader understanding of the environmental factors that may have shaped the adaptation of wheat species during domestication, and we believe changes in N supply are one important driving force.

L324-355 this section is largely speculative, and I suggest reducing it substantially. The whole discussion about pathogen resistance, or lysine, SACPATH etc, are potentially interesting, but are not supported by experimental evidence sufficient to warrant such a detailed discussion. I think that authors should make a further effort to avoid chasing those results that support their hypothesis, but rather stick to the description of the findings (that are already interesting enough without the need of speculation)

Response: We appreciate the reviewer's perspective and acknowledgment of our findings. We believe that while it is important to maintain a balance between speculation and evidence-based findings, the aspects that we discuss are grounded in existing literature and provide valuable context and potential implications of our results. We are of the opinion that this detailed discussion enhances the overall contribution of our work, and therefore, we would prefer to retain this section in its current form.

L326 here and elsewhere, whenever "enrichment" is reported, add p values

Response: The p-values are always specified in the dedicated Supplemental Figures and/or Datasets.

L522 remove "pioneering" and all claims about the exceptionality of this study. It is up to the reader to assess this.

Response: While we understand the suggestion to remove the term "pioneering" and claims about the exceptionality of this study, we believe that these descriptors serve to highlight its unique contributions and provide valuable context for readers. It is important to note that we also acknowledge the limitations of the study and areas for potential improvement. We have ensured that these claims are presented in a balanced manner to allow readers to form their own assessments.

L527 is this sentence missing a verb?

Response: We have now corrected this sentence (lines 598-601).

L794 this is nice, and reinforces the methodological ambition of this manuscript

Response: We thank the reviewer for this feedback.

***Reviewer #2**:*

In the resubmitted paper, the authors analyzed the impact of domestication on transcriptome plasticity under nitrogen starvation in durum wheat. The most significant change in the article is the additional analysis, which demonstrates the selection of the bread wheat genome as the optimal reference for the analysis. Overall, I believe that my queries have been addressed as well.

I have two additional question:

The authors applied several arbitrary filters to the gene expression data prior to the QST-FST analysis, reducing the number of analyzed genes to 5,868. However, the population background used in the gene ontology analysis is not specified. I assume that all annotated wheat genes were used as the background population. It is possible that the categories found to be over represented in the 967 genes that satisfied the QST-FST analysis criteria are already over represented in the 5,868 analyzed genes. Can the authors specify the population background used for the analysis? This information could have a significant impact on the results.

Response: In the Materials and Methods section we previously reported "*Enriched terms in the DEGs and genes under selection were identified using agriGO v.2.0 (Tian et al., 2017) with T. aestivum reference annotations*".

To make it more explicit we have now modified the sentence "*Enriched terms in the DEGs and genes under selection were identified using agriGO v.2.0 (Tian et al., 2017). All T. aestivum's annotated genes were used as background…*". (lines 798-799).

The choice of the background set indeed influences the interpretation of significantly overrepresented GO categories. If certain categories are already overrepresented in the 5,868 genes due to the initial filtering, using this set as the background might mask

true biological insights. Using all annotated genes of the organism (in this case, wheat) as the background, unless there is a specific and justified reason to use a different set, prevents biasing the GO enrichment analysis toward certain functions or categories. Therefore, using the entire annotated genome was the only option considered for our work.

Several values of Qst in Data S4 have value 0 or 1. How this can be interpreted?

Response: In the Supplemental Data Set S4 we provided Q_{ST} values for each gene identified as being under selection in at least one of the six groups (3 pairwise subspecies comparisons x 2 N conditions) following our “selection scan”. This means that a gene having a value of Q_{ST} falling in the 5% right-hand tail of one of the six distributions (and thus classified as “under selection” in that specific comparison and N availability context), might have a lower, nonsignificant value in one or more of the other groups. Therefore, the same gene can exhibit different values, ranging from 0 to 1, across the various groups.

***Reviewer #3**:*

I appreciate that the authors considered my and other reviewers' suggestions and comments in the manuscript revision, especially their efforts in reanalyzing using wild emmer and durum wheat as reference genomes. However, I feel the re-analyses have not been fully incorporated into the revised manuscript beyond some verbal justification. For example, in the revised manuscript, lines 153-166, the authors claimed that they had corrected the reference bias by mapping reads to tetraploid wheat reference genomes, but they failed to mention any numerical or statistical evidence to support why "bread wheat was the best choice" other than citing the Supplemental datasets. Also, these supplemental datasets should be visualized either as main figures or supplementary figures. Otherwise, it is hard to understand why the reference bias wouldn't affect the interpretation of the results.

Response: In the revised manuscript, we aimed to keep the main text concise by including the re-analysis details in a "Supplemental Method" section. However, we understand the importance of fully incorporating these results into the main text to address concerns about reference bias. In response to the reviewer's feedback, we have now included a dedicated paragraph discussing the choice of the reference genome in the Results and Discussion section. Additionally, we incorporated results from using different reference genomes in the section on nucleotide diversity.

We are of the opinion that the Supplemental Datasets provide a suitable means to present the detailed mapping statistics and variant numbers. These datasets are extensive and detailed, and their inclusion in the main text could compromise readability. We have provided instead a more detailed explanation in the main text to aid understanding. The Supplemental Datasets remain available for transparency and completeness, ensuring that all our data is accessible.

I expect that genetic distances (such as using the IBS approach) will be calculated pairwise and used to justify why a certain reference would be biased and how to interpret the results accordingly. I do not completely follow what the Mash software is doing and why they calculate the average nucleotide identity. What this average nucleotide identity means is not clearly explained.

Response: Since we needed to present the distances among the three available reference genomes (*Triticum aestivum* cv. Chinese Spring, *Triticum turgidum* ssp. *dicoccoides* accession Zavitan, *Triticum turgidum* ssp. *durum* cv. Svevo), using an approach based on variant calling, such IBS, was not applicable. Instead, we opted to use the Mash software, which employs an alignment-free method based on MinHash. Mash compresses large genomic sequences into sketch representations, enabling rapid similarity estimations with bounded error. The Mash distance is an approximation of the mutation rate and from these computed distances, the average nucleotide identity (ANI) can be derived. ANI provides a measure of the genomic similarity between two sequences, offering insight into their genetic relatedness. We recognize that this explanation was not clearly presented in the revised text. Therefore, we have now added a more detailed description of the methodology in the additional paragraph. This should clarify why we used this approach and how to interpret the results accordingly.

In lines 171-174, the authors simply conducted site frequency spectra analysis without any interpretation. I would expect further statistical analysis or comparison by partitioning the SFS into genomic features, such as 5'UTR, exonic, or intronic regions, etc.

When addressing my comments regarding 'private' or 'shared' SNPs, the authors provided a definition as requested. However, I believe there is an opportunity here for a more in-depth analysis. A deeper exploration of 'private' SNPs, if not due to SNP calling error, could potentially reveal the relationship between selection and genetic drift. As hypothesized by Beissinger et al. 2016 in a maize study, low-frequency (such as singleton) or younger polymorphisms might show distinct patterns from pairwise diversity, which is mainly determined by intermediate frequency or older alleles. A comprehensive analysis and comparison with what has been discussed in maize and other species could significantly enhance the paper's strength.

Response: While we recognize the potential value of the suggested additional analysis, regarding a deeper characterization of SFS and exploration of private SNPs, we believe that they are not relevant in the context of our work.

Our focus remains on presenting the core findings related to gene expression plasticity and selection signatures under diverse nitrogen availability conditions during tetraploid wheat domestication, while also leveraging transcriptomic data to investigate nucleotide diversity beyond conventional differential expression analysis. Such specific analysis as suggested by the reviewer would require a substantial shift in our research objectives. Moreover, we are not sure that our type of data (RNA-seq) would

be suitable for such a deep analysis as described in the work of Beissinger et al. (2016), where whole genome sequencing data were employed.

We appreciate the reviewer's suggestions; however, our current dataset and research goals were not designed to support such extensive comparative analysis. Thus, we have opted to keep our analyses focused and concise concerning the nucleotide diversity analysis. We hope our response clarifies the rationale behind our decision.

Dear Dr. Papa:

We are pleased to inform you that your paper entitled "Transcriptomic Response to Nitrogen Availability Highlights Signatures of Adaptive Plasticity During Tetraploid Wheat Domestication" has been accepted for publication in The Plant Cell, pending a final editorial review by a science editor. At this stage, your manuscript will be evaluated by a science editor with respect to its presentation of scientific content, compliance with journal policies, and presentation for a broad readership. The Plant Cell has contracted with Plant Editors (planteditors.com) to provide this service to our authors, and you will soon receive additional information on this process.

Please note that each author needs to link their ORCID identifier to their account in the system before your manuscript can be published. If any authors do not have an ORCID linked to their account, they will receive a message with a link to complete this task. Please ensure that ALL of your coauthors have completed this task as soon as possible.

ASPB offers an OPEN option that allows authors to have their online articles available for free to all users immediately upon publication. For more information about the ASPB OPEN option, refer to the Final Submission Checklist Form.

The Plant Cell and The Arabidopsis Information Resource (TAIR) are collaborating to collect functional annotation data about Arabidopsis genes from authors. This includes information about the gene's molecular function (e.g., kinase activity, ATP synthetase activity), the biological process/es it is involved in (e.g., endosperm development, threonine biosynthesis), its subcellular location (e.g., nucleus, ER), anatomical or developmental expression pattern (e.g., leaf, ovule, flower stage 10, seedling stage), or its partner in a protein-protein interaction (e.g., AT1G01010 interacts with AT1G01020).

If your paper contains results falling into one or more of these categories for Arabidopsis genes, we request that you now submit these data for inclusion in TAIR by filling in the form provided at the following URL: https://www.arabidopsis.org/doc/submit/functional_annotation/123. If you need further clarification on what types of data can be submitted please contact curator@arabidopsis.org.

Finally, we encourage your submission of artwork for the journal cover. Monthly issues will have an online cover image and selected covers will be used for posters, other promotional items, and "wallpaper" for mobile devices. For more information, refer to Cover Submission in the Instructions for Authors https://tpc.msubmit.net/cgi-bin/main.plex?form_type=display_auth_instructions.

We look forward to seeing your paper published.

Sincerely,

The Plant Cell Board of Editors

=====
IMPORTANT REMINDER: PEER REVIEW REPORTS
=====

If you opted to publish a peer review report along with your article during the original submission process, it will be prepared by the editorial staff and publicly posted with your manuscript, inside the zip file that contains any other supplemental material. As a reminder, the peer review report is a public record of all comments from editors and reviewers, as well as your prior responses, as you received them in the decision letters for each draft of your manuscript. If you agreed to publish this report and have changed your mind, or are not sure if you selected this option, please contact the editorial office as soon as possible before signing the license agreement from our publisher.

=====

----FOR ASPB OFFICE USE ONLY (DO NOT EDIT)----
MSID: 36468
Corresponding Author: Prof. Papa
